# Human CD6 Down-Modulation following T-Cell Activation Compromises Lymphocyte Survival and Proliferative Responses

**DOI:** 10.3389/fimmu.2017.00769

**Published:** 2017-06-30

**Authors:** Esther Carrasco, Cristina Escoda-Ferran, Núria Climent, Cristina Miró-Julià, Inês T. Simões, Mario Martínez-Florensa, Adelaida Sarukhan, Esther Carreras, Francisco Lozano

**Affiliations:** ^1^Grup d’Immunoreceptors del Sistema Innat i Adaptatiu, Institut d’Investigacions Biomèdiques August Pi i Sunyer (IDIBAPS), Barcelona, Spain; ^2^IDIBAPS-AIDS Research Group, HIVACAT, Barcelona, Spain; ^3^Institut National de la Santé et de la Recherche Médicale (INSERM), Paris, France; ^4^Servei d’Immunologia, Hospital Clínic de Barcelona, Barcelona, Spain; ^5^Departament de Biomedicina, Facultat de Medicina, Universitat de Barcelona, Barcelona, Spain

**Keywords:** lymphocyte activation, CD6, T cells, apoptosis, surface receptor down-modulation, T-cell homeostasis

## Abstract

Available evidence indicates that the CD6 lymphocyte surface receptor is involved in T-cell developmental and activation processes, by facilitating cell-to-cell adhesive contacts with antigen-presenting cells and likely modulating T-cell receptor (TCR) signaling. Here, we show that *in vitro* activation of human T cells under different TCR-ligation conditions leads to surface downregulation of CD6 expression. This phenomenon was (i) concomitant to increased levels of soluble CD6 (sCD6) in culture supernatants, (ii) partially reverted by protease inhibitors, (iii) not associated to CD6 mRNA down-regulation, and (iv) reversible by stimulus removal. CD6 down-modulation inversely correlated with the upregulation of CD25 in both FoxP3^−^ (T_act_) and FoxP3^+^ (T_reg_) T-cell subsets. Furthermore, *ex vivo* analysis of peripheral CD4^+^ and CD8^+^ T cells with activated (CD25^+^) or effector memory (effector memory T cell, CD45RA^−^CCR7^−^) phenotype present lower CD6 levels than their naïve or central memory (central memory T cell, CD45RA^−^CCR7^+^) counterparts. CD6^lo/−^ T cells resulting from *in vitro* T-cell activation show higher apoptosis and lower proliferation levels than CD6^hi^ T cells, supporting the relevance of CD6 in the induction of proper T-cell proliferative responses and resistance to apoptosis. Accordingly, CD6 transfectants also showed higher viability when exposed to TCR-independent apoptosis-inducing conditions in comparison with untransfected cells. Taken together, these results provide insight into the origin of sCD6 and the previously reported circulating CD6-negative T-cell subset in humans, as well as into the functional consequences of CD6 down-modulation on ongoing T-cell responses, which includes sensitization to apoptotic events and attenuation of T-cell proliferative responses.

## Introduction

Immune modulation is an active area of medical research for development of novel therapies that apply to immune-mediated disorders. Most promising targets are co-stimulatory (e.g., CD28) and—inhibitory (e.g., CTLA-4) immunoreceptors present at the immune synapse (IS)—the site of antigen-presenting cell (APC)–T-cell interaction that drives T-cell activation. CD6 is lymphocyte surface accessory molecule associating to the T-cell receptor (TCR) complex at the center of the IS (1), whose modulatory role (either positive or negative) in T-cell activation has not been definitively established, in part due to the lack of suitable genetically modified animal models. This contrasts with the identification of CD6 as a significant susceptibility locus for multiple sclerosis in genome-wide association studies (2) and the promising results of treating some autoimmune disorders with non-depleting anti-CD6 mAb (3).

CD6 is a 105–130 kDa glycoprotein expressed by all thymocytes and mature T cells, a subset of NK and B cells (4, 5), and some hematopoietic cell precursors (6), and brain areas (7). It is composed of three scavenger receptor cysteine-rich (SRCR) extracellular domains, a transmembrane region and a cytoplasmic tail without intrinsic catalytic activity but harboring several motifs susceptible of phosphorylation and/or interaction with signal-transducing effectors (8). Notably, CD6 plays a key role in the stabilization and maturation of the IS, as well in subsequent T-cell proliferative responses (9–11). This is achieved through interaction of membrane-proximal SRCR domain (D3) of CD6 with the N-terminal domain (D1) of CD166/activated leukocyte cell adhesion molecule (ALCAM), a broadly distributed adhesion molecule of the Ig superfamily, expressed by dendritic cells (DC), macrophages, B cells, and thymic epithelial cells among others (12, 13). The CD6 signaling pathway is only partially known and includes the activation of MAPK (14), and recruitment of Syntenin-1 (15) and SH2 domain-containing leukocyte protein of 76 kDa (SLP-76) (16). Recently, CD6 has been identified as a critical protein in the linker for the activation of T cells (LAT)-independent TCR signaling scaffold (17).

Changes in CD6 surface levels have been reported under both physiologically and experimentally induced conditions, but their ultimate significance is not fully understood. In thymus, CD6 expression levels seem to be developmentally regulated as they increase when immature double-positive thymocytes are selected to a mature single-positive stage (18). CD6 upregulation on both immature and mature thymocytes but not PBL is efficiently achieved *via* CD2 compared to CD3 cross-linking and correlates well with resistance to apoptosis, suggesting that CD6-dependent signals contribute to the positive selection and survival of thymocytes (18). Protein kinase C (PKC) activators also positively upregulate CD6 expression on both peripheral T cells and thymocytes but only negligibly on lymphoblastoid B-cell lines (19). Studies in herpes virus-infected patients showed a reduced expression of CD6, CD28, and CD5 on CD8^+^ T cells that expand after viral infection, while the CD4^+^ T cells maintained normal levels of these markers (20). Interestingly, such a CD8^+^ T-cell population was more susceptible to both spontaneous and activation-induced cell death (AICD) (20). An intriguing finding was also the discovery of a small subset of peripheral blood T cells from healthy subjects (5–6% of CD3^+^ cells) expressing little or no CD6 (CD3^+^CD5^int^CD6^lo/−^) in both the CD4^+^ and CD8^+^ subsets (21). These CD6^lo/−^ T cells were reported as of unknown precursor origin and showed substantially reduced reactivity to allogeneic stimulation but not to mitogenic lectins (PHA) or soluble recall antigens (tetanus toxoid) (21). Recently, it has been reported that human natural regulatory T cells (nT_reg_) are characterized by low/negative expression of CD6 (22), and the authors hypothesized that it could be the same CD6^lo/−^ population identified by Rasmussen et al. (21).

The existence of a soluble CD6 (sCD6) form of unknown origin and circulating at very low levels (pico/nano molar range) in sera from healthy individuals has also been reported (23). Interestingly, elevated sCD6 levels were observed in patients suffering of primary Sjögren’s syndrome (24) and systemic inflammatory response syndrome (25), although a direct mechanistic and/or functional relationship between the two events is lacking.

The present study analyzes CD6 expression following different *in vitro* T-cell stimulatory conditions. The results reported herein indicate that CD6 is down-modulated in part due to proteolytic cleavage, and this contributes to homeostatic control of ongoing immune responses by making T cells more prone to apoptosis and less responsive to proliferative stimuli.

## Materials and Methods

### Cells

PBL were obtained by standard Ficoll gradient (*d* = 1,077) and further depletion of plastic-adherent cells from peripheral blood of healthy volunteers from the Banc de Sang i Teixits of Catalonia. Written informed consent was obtained from all donors. The study was carried out according to the recommendation and approval of the Ethics Committee of Clinical Investigation from the Hospital Clinic of Barcelona. The human Jurkat T-lymphoma cell line was from the American Tissue Culture Collection. The parental and CD6-transfected 2G5 Jurkat derivative cells (CD5^−^ and CD6^−^) were generated as reported elsewhere (14, 26). Unless otherwise indicated, cells were cultured in RPMI 1640 (Lonza) medium supplemented with 10% FBS (Gibco) and gentamicin (0.5 μg/mL, Braun).

### Antibodies and Reagents

The anti-CD6 161.8 (IgG_1_) and OX124 (IgG_1_) mAbs were kindly provided by Ramón Vilella (Immunology Department, Hospital Clínic, Barcelona) and Steven Cobbold (University of Oxford, Oxford, UK), respectively. Recombinant soluble human CD6 (rshCD6) and CD5 (rshCD5) were produced as previously reported (9, 23). Staphylococcal enterotoxin B (SEB) was from Sigma.

### Flow Cytometry Analyses and Cell Sorting

Human PBL were suspended in PBS containing 2% FBS for 30 min on ice and, then, stained with different combinations of fluorochrome-labeled mAb: CD6-FITC (M-T605), CD4-FITC (L200), CD45RA-PerCP-Cy™5.5 (HI100), and CD25-APC-H7 (M-A251) from BD Pharmingen; CD3-APC-Cy™7 (SP34-2), CD3-PE (UCHT1), CD25-V450 (M-A251), IgG1k-PE (MOPC-21), IgG1k-APC (MOPC-21), and CD197/CCR7-PE (3D12) from BD Biosciences; CD3-APC (MEM-57) and CD25-APC (MEM-181) from Immunotools; CD8-APC (HIT8a), CD8-Pacific Blue™ (SK1), CD27-Pacific Blue™ (M-T271), CD178/FasL-PE (NOK-1), and CD95/Fas-PerCP-Cy5.5 (DX2) from Biolegend; CD4-Alexa Fluor^®^647 (MT310) from Santa Cruz Biotechnology; CD5-PerCP-Cy5.5 (UCHT2) from TONBO Biosciences; and goat anti-mouse Ig-FITC from Sigma. T_reg_ cells were detected by intracellular FoxP3 staining kit (BD Pharmingen) following manufacturer’s instructions. Cell viability and apoptosis assays were performed by using Live/Dead Fixable Violet Dead stain kit (Life Technologies), Annexin V-FITC (Apoptosis Detection kit; Life technologies) or Annexin V-PE (BioLegend) and 7-AAD (BD Pharmingen), following manufacturer’s instructions. Cells were run on a FACS Canto II instrument (BD Bioscience) and analyzed by FlowJo software.

For cell sorting purposes, PBL (5 × 10^6^ cells) was harvested at day 3 post-PHA activation (2 μg/mL; Sigma), suspended in PBS plus 2% FBS and 2 mM EDTA, and then labeled for CD3, CD6, and CD25 expressions. Nonviable cells were discarded using Live/Dead Fixable Violet Dead stain kit. The desired T-cell subsets (CD3^+^CD25^+^CD6^hi^) and (CD3^+^CD25^+^CD6^lo/−^) were sorted on a FACSAria III machine (BD Bioscience), further stained with Cell trace™ CFSE Cell Proliferation kit (Life Technologies), and cultured for an additional 6 days. At days 2 and 6, cells were analyzed for apoptosis and proliferation.

### T-Cell Stimulation Assays

PBL (10^5^ cells/well) were cultured with PHA (5 μg/mL), anti-CD3 plus anti-CD28 (αCD3/CD28) dynabeads (2 μL/10^5^ cells; Life Technologies), PMA (10 ng/mL; Sigma), or rhIL-2 (10 ng/mL; Roche) in X-VIVO™15 medium (Lonza) for 4 days at 37°C. In some experiments, at day 3 of culture, cells were stimulus-deprived and then re-stimulated with PHA (1 μg/mL) or left untouched for an additional 13-day period. Protease inhibitor TAPI-2 (100 mM; Sigma) or Complete™ (Roche) effects were tested on PHA-activated (2 μg/mL) PBL.

For T-cell proliferation assays, PBLs (10^5^ cells/well) were labeled with CFSE and co-cultured with 2 × 10^3^ SEB preloaded mature DCs (mDCs) from an unrelated donor and generated as described elsewhere (27). In some experiments, at day 5, cells were analyzed for CD6 surface expression with biotinylated 161.8 mAb (2 μg/mL) plus Streptavidin (SAv)-Alexa Fluor^®^ 700 (Invitrogen). Percent of proliferating cells and number of cell cycles were determined by measuring the geometric mean of fluorescence intensity (Geo MFI) channel of CFSE^lo^ and CFSE^hi^ cells by flow cytometry and the FlowJo software.

### Detection of sCD6

ELISA detection of human sCD6 was performed on 96-well microtiter plates (Nunc, Roskilde, Denmark) coated overnight (o/n) at 4°C with PBL supernatants (SN) (100 μL). After blocking for 1 h at room temperature (RT) with PBS plus 3% BSA, biotinylated 161.8 mAb (2 μg/mL) was added and incubated for 2 h at RT. Bound mAb was detected by 1 h incubation at RT with SAv-Peroxidase (SAv-POD; Roche) and further color development by the addition of 3,3′,5,5′-tetramethylbenzidine liquid substrate (Sigma). Absorbance was measured at 450 nm with Epoch spectrophotometer (BioTek).

For immunoprecipitation of sCD6, protein G sepharose beads (GE Healthcare Life Sciences) were coupled to 161.8 mAb following manufacturer’s instructions and, then, incubated o/n at 4°C under orbital rotation with 1 mL SN from PHA- or rhIL-2-treated PBL. RshCD5 (500 ng) and rshCD6 (500 ng) were used as negative and positive controls, respectively. After washings, beads were eluted and run on 8% SDS-PAGE under reducing conditions for further Western blot analysis with rabbit anti-human CD6 antiserum (1:500) produced in our laboratory (27), followed by HRP-labeled donkey anti-rabbit Igs (GE Healthcare Life Sciences). Chemiluminescence was developed with Supersignal West Dura Extended Duration Solution (Pierce, Rockford, IL, USA) and images were obtained using ImageQuant LAS 4000 (GE Healthcare Life Sciences).

### Western Blot Analysis

Cells were lysed after a 30 min incubation on ice with lysis buffer (10 mM Tris-HCl pH 7.6, 140 mM NaCl, 5 mM EDTA, 140 mM NaF, 0.4 orthovanadate, 5 mM pyrophosphate, 1 mM phenylmethylsulfonyl fluoride, protease inhibitor mixture tablets (*Complete*™), and 1% non-idet P-40). After centrifugation at 12,000 × *g* for 15 min at 4°C, *Laemmli*’s buffer was added to 50 μL from cell lysates. Samples were boiled and run on 8% SDS-PAGE under reduction conditions for further WB analysis. Membranes were incubated with rabbit anti-human CD6 poAb (1:500), followed by anti-rabbit-HRP (1:2,500) (Roche). Chemiluminescence was developed with Supersignal West Dura Extended Duration Solution (Pierce, Rockford, IL, USA). Images were obtained using LAS 4000, and bands were quantified with Photoshop CS5 software.

### Quantitative Reverse Transcription PCR (RT-qPCR) Assays

Total RNA from non-treated or 24 h treated with rhIL-2 (10 ng/mL), PHA (5 μg/mL), or PMA (10 ng/mL) PBMC cells was obtained with PureLink RNA Mini Kit (Ambion, Life technologies) according to manufacturer’s instructions. RNA was retrotranscribed into cDNA using the High capacity cDNA Reverse Transcription kit (Thermofisher). RT-qPCR was performed on an Applied Biosystems 7900 HT Fast Real Time PCR System using Taqman Fast universal PCR master Mix (Life Technologies/ThermoFisher) and CD6 specific TaqMan expression assays (Hs00198752_m1: exon boundary 1–2) and (Hs00945709_m1: exon boundary 5–6) (ThermoFisher Scientific). Results in triplicates were normalized to gycleraldehyde-3-phosphate dehydrogenase (GAPDH, Hs99999905_m1), using the 2ΔCt formula, where ΔCt = Ct (GADPH) − Ct (gene of interest). Results are represented as relative values.

### Cytotoxicity Assays

Parental and CD6-transfected 2G5 cells were treated with different concentrations of doxorubicin (0.1–10 μM, Pfizer), PHA (2 μg/mL), or puromycin (0.25–2.5 μg/mL) for 24–48 h at 37°C in culture medium and, then, analyzed by flow cytometry for apoptosis cell detection with Annexin V-FITC or Annexin V-PE and 7-AAD.

### Statistical Analyses

Data are expressed as the mean ± SD or SEM using GraphPad software. Significant differences between two groups were determined by using unpaired Student’s *t*-test and between three or more groups with one-way ANOVA followed by Tukey’s test.

## Results

### CD6 Surface Expression Is Downregulated following T-Cell Activation

**Figure 1:**
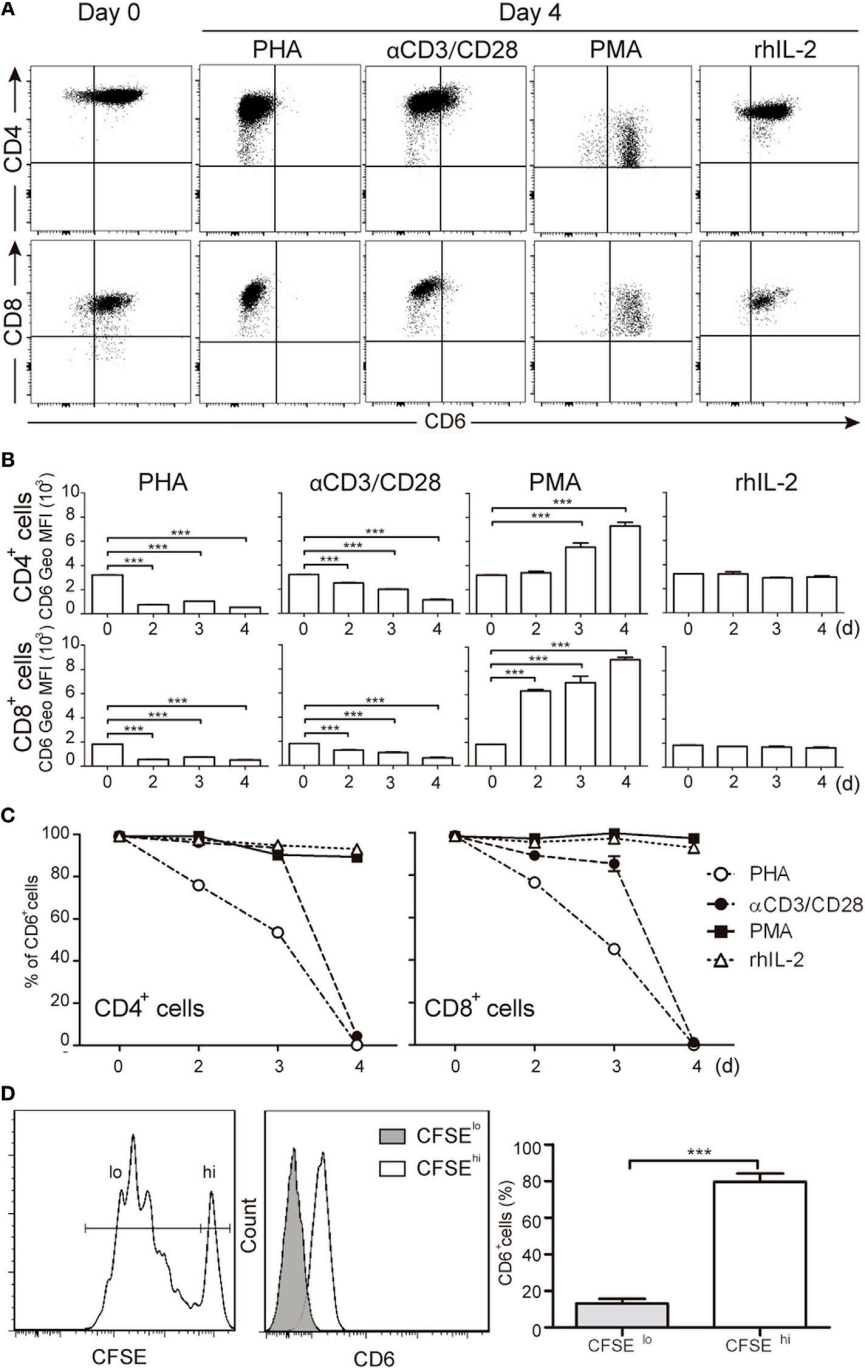
Downregulation of CD6 surface expression in response to different T-cell receptor-mediated activation stimuli. **(A–C)** PBLs (10^5^/well) were cultured for 4 days in the presence of PHA (5 μg/mL), αCD3/CD28 beads (2 μL/10^5^ cells), PMA (10 ng/mL), or rhIL-2 (10 ng/mL). Cells were then stained with labeled specific mAbs for CD3 (UCHT1), CD4 (MT310), CD8 (SK1), and CD6 (M-T605) and analyzed by flow cytometry at different days (d). **(A)** Representative dot plots of CD6^+^ cells in the CD3^+^CD4^+^ (top panel) or CD3^+^CD8^+^ (bottom panel) subsets at days 0 and 4 post-stimulation. **(B)** Bar chart representing mean ± SD of geometric mean of fluorescence intensity (Geo MFI) values for CD6 expression on CD3^+^CD4^+^ (top panel) and CD3^+^CD8^+^ (bottom panel) cells over time post-stimulation, from three independent experiments. **(C)** Line chart representing the percentage of CD6^+^ cells over time post-stimulation in the CD3^+^CD4^+^ (left hand) or CD3^+^CD8^+^ (right hand) subsets from three independent experiments. **(D)** Staphylococcal enterotoxin B-pulsed mature DCs (2 × 10^3^/well) co-cultured with CFSE-labeled PBL (10^5^/well) for 5 days were analyzed for CD6 expression on proliferating (CFSE^lo^) and non-proliferating (CFSE^hi^) cells. Left, representative histogram of CD6 expression on CFSE^lo^ and CFSE^hi^ cells is. Right, bar chart representing percent (mean ± SEM) of CD6^+^ cells in the CFSE^lo^ and CFSE^hi^ subsets from 10 independent experiments. ***p < 0.001 (unpaired *t* test).

CD6 is highly expressed on major human T-cell subsets (CD3^+^CD4^+^ and CD3^+^CD8^+^) from resting PBLs (Figures 1A–C). However, following TCR-mediated activation with either PHA (5 μg/mL) or anti-CD3 plus anti-CD28 beads (αCD3/CD28) a significant and sustained reduction of CD6 surface levels was observed in both peripheral T-cell subsets compared to unstimulated cells cultured in the presence of rhIL-2 (Figures 1A–C). As expected from previous reports (18), CD6 expression was upregulated by PMA (10 ng/mL), a potent polyclonal T-cell activator overcoming the need for TCR-ligation (Figures 1A,B). Since the T-cell activating agents used above (PHA, PMA, and αCD3/CD28) might result in supra-physiological responses, changes in CD6 surface expression were analyzed following a more physiological cell contact-dependent TCR stimulation system. To this end, CFSE-labeled PBL was co-cultured for 5 days with SEB pre-loaded allogeneic mDCs and proliferating (CFSE^lo^) and non-proliferating (CFSE^hi^) cells were analyzed for CD6 expression. As illustrated by Figure 1D, CFSE^lo^ cells showed a significant reduction of CD6 expression, as measured by Geo MFI, compared to CFSE^hi^ cells. These results support the notion that CD6 downregulation is an integral late event of TCR-mediated T-cell activation. Furthermore, CD6 downregulation was specific since the expression of CD5, a closely related lymphoid receptor partly associating with CD6 (28), was upregulated under the same stimulatory conditions (Figure S1 in Supplementary Material), according to previously reported data (29).

### CD6 Expression Inversely Correlates with CD25 Expression following T-Cell Activation Regardless of FoxP3 Induction

**Figure 2:**
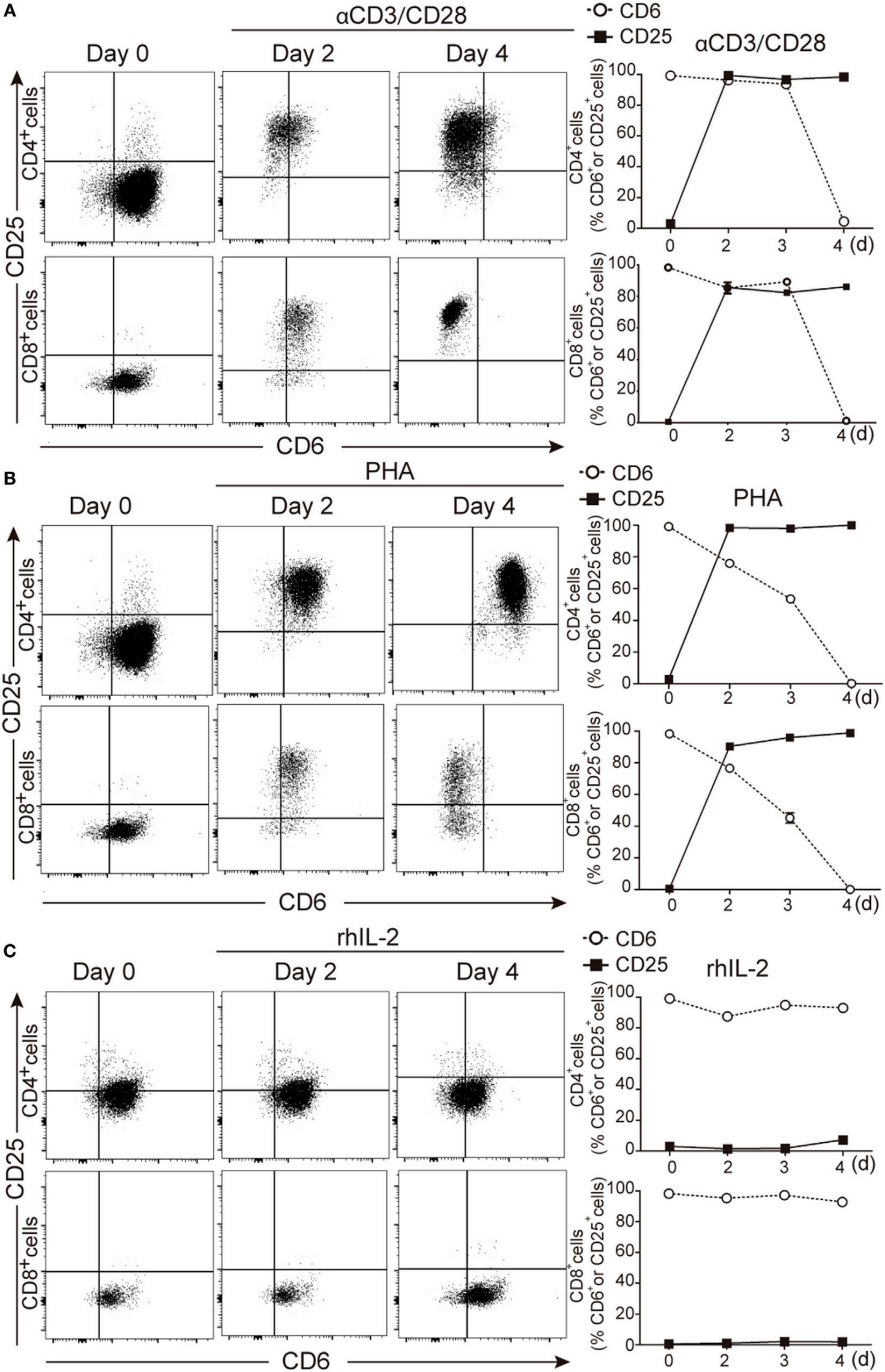

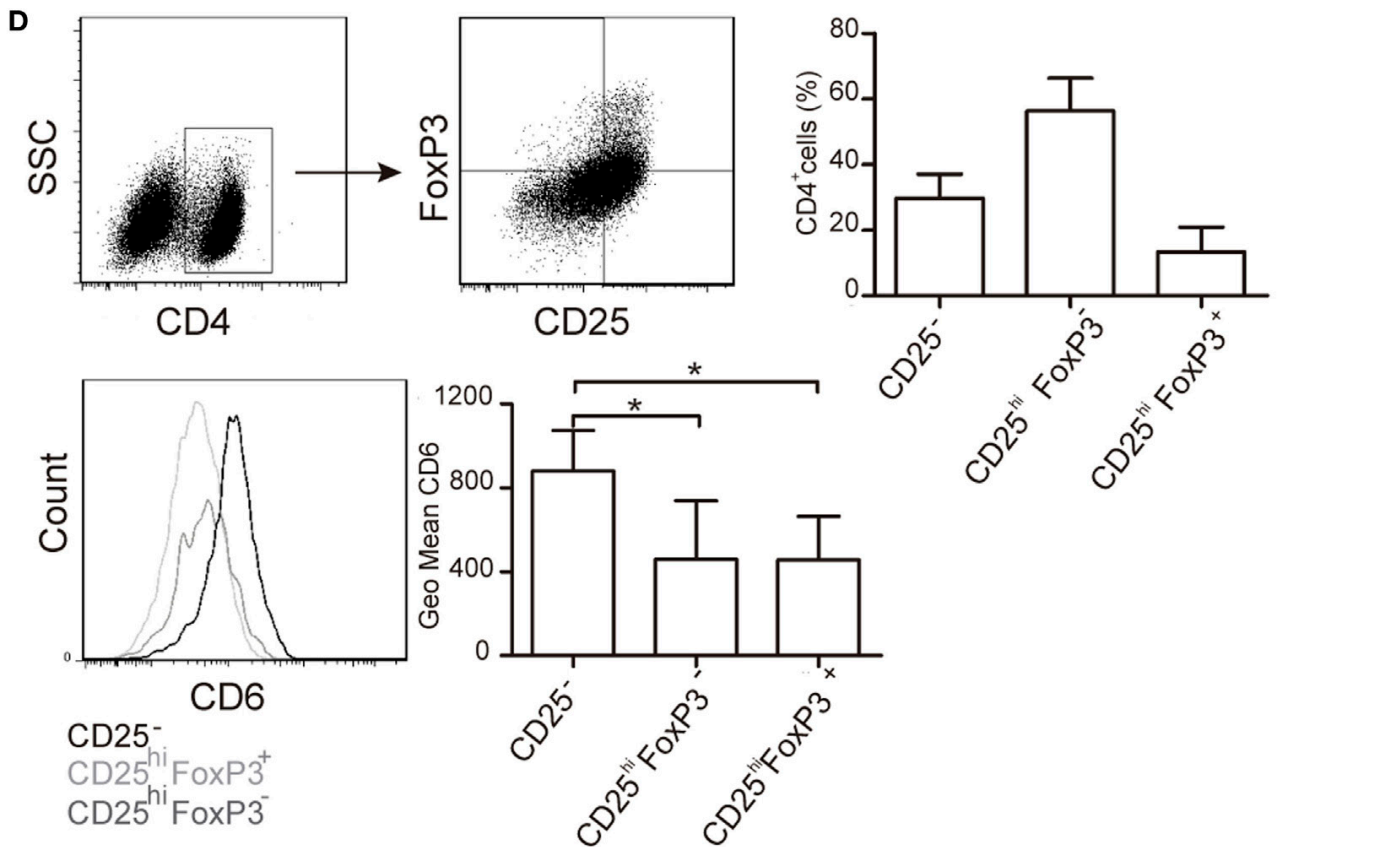
Downregulation of CD6 surface expression on CD25^+^ cells following T-cell activation. **(A–C)** PBLs (10^5^/well) cultured in the presence of αCD3/CD28 (2 μL/10^5^ cells) PHA (2 μg/mL) or rhIL-2 (10 ng/mL) for 4 days, were stained with labeled CD3 (UCHT1), CD4 (MT310), CD8 (SK1), CD5 (UCHT2), CD6 (M-T605), and CD25 (M-A251) mAbs for flow cytometry analysis at different time points. **(A)** Left, representative dot plot of CD6 and CD25 expression over time on CD4^+^ (top panel) and CD8^+^ (bottom panel) cells upon αCD3/CD28 stimulation. Right, line chart representing the percent of CD6^+^ or CD25^+^ cells in CD4^+^ (top panel) or CD8^+^ (bottom panel) cells over time. **(B)** The same as in **(A)** but upon culture of cells with PHA (2 μg/mL). **(C)** The same as in **(A)** but upon culture of cells with rhIL-2. Data represented in **(A–C)** are the mean ± SD from three independent experiments. **(D)** PBL (10^5^/well) stained for surface CD4, CD25, and CD6 (see above) and intracellular FoxP3 before and after exposure to PHA (2 μg/mL) for 4 days were analyzed by flow cytometry. Top left, representative dot plot of the gating strategy used. Top right, bar chart showing the percent of CD4^+^ cells with CD25^−^, CD25^hi^FoxP3^−^, and CD25^hi^FoxP3^+^ phenotype. Bottom left, representative histogram of CD6 surface levels on CD25^−^, CD25^hi^FoxP3^−^, and CD25^hi^FoxP3^+^ T-cell subsets. Bottom right, bar chart showing the mean ± SD of geometric mean of fluorescence intensity values for CD6 expression of triplicates from two experiments. *p < 0.05 (unpaired *t* test).

To further confirm that downregulated CD6 expression was linked to T-cell activation, PBLs were double stained for CD25 and CD6 expression at different time points following stimulation with αCD3/CD28 beads, PHA (2 μg/mL) or rhIL-2 (10 ng/mL). As shown in Figures 2A–C, both CD4^+^ and CD8^+^ T cells increased their CD25 expression post-stimulation with αCD3/CD28 or PHA and concomitantly decreased that of CD6 at later time points (96 h in the represented case). It is noteworthy that CD6 down-modulation could be detected at even earlier time points depending on the donor and the use of high PHA doses (5 μg/mL) (Figure S2 in Supplementary Material).

Next, CD6 downregulation was explored in different activated CD4^+^ PBL subsets (CD25^hi^FoxP3^−^ and CD25^hi^FoxP3^+^) resulting from PHA stimulation. As illustrated by Figure 2D, lower CD6 surface levels were observed for CD25^hi^ cells, irrespective of their FoxP3 phenotype, compared to unstimulated naïve CD25^−^ cells. This indicates that CD6^lo/−^ expression is characteristic of CD25^hi^ T cells but do not strictly correlate with FoxP3 expression.

### CD6 Expression Is Downregulated on Effector but Not Central Memory T-Cells

**Figure 3:**
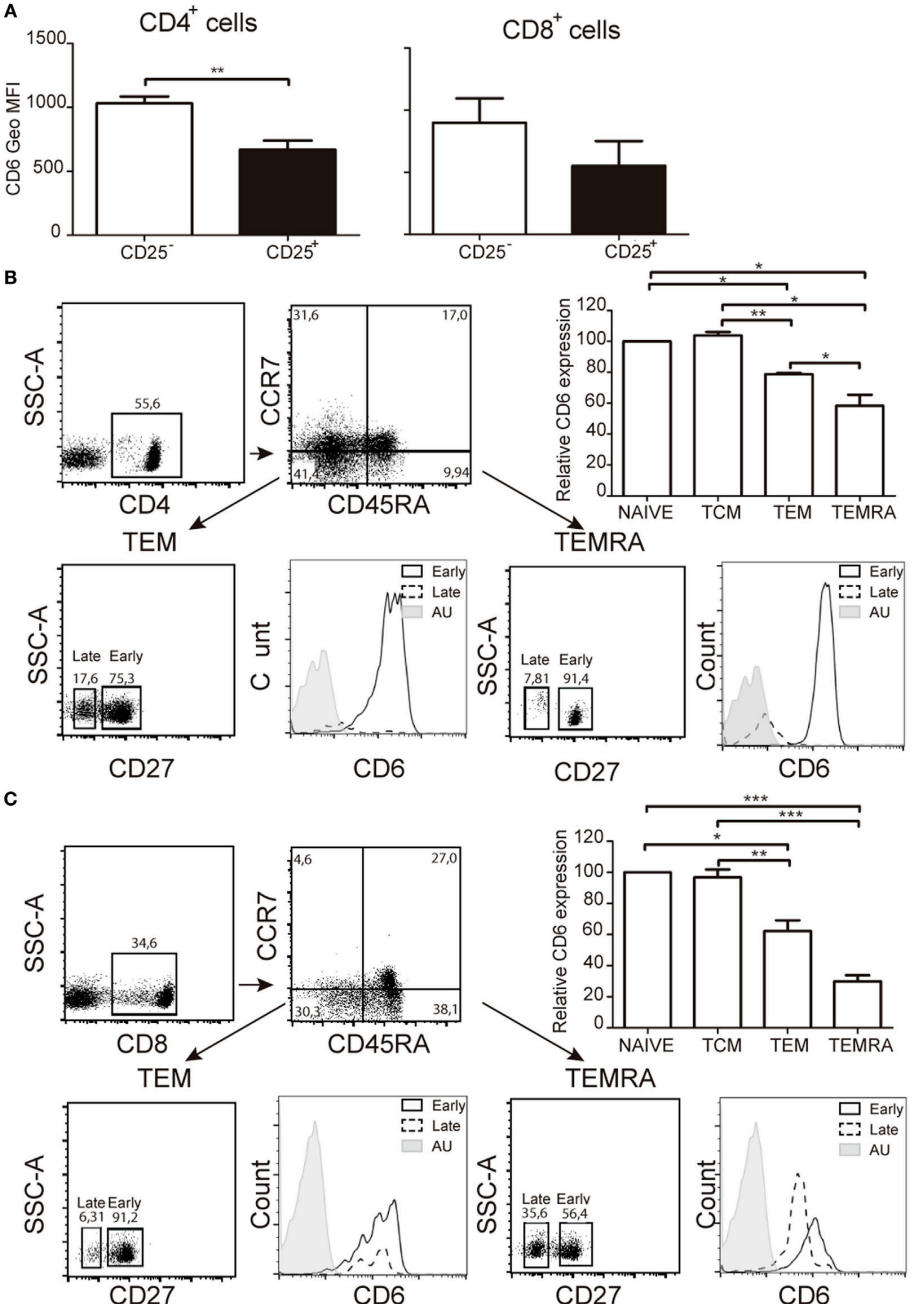
*Ex vivo* analysis of CD6 surface expression on peripheral T cells with activated or memory phenotypes. **(A)** Bar chart representing geometric mean of fluorescence intensity (Geo MFI) (mean ± SEM) of CD6 surface expression on CD25^−^ and CD25^+^ peripheral CD4^+^ (left hand side) and CD8^+^ (right hand side) T cells from healthy donors (*n* = 4). **(B)** Analysis of CD6 surface expression of peripheral CD4^+^ T cells from healthy donors with naïve (CD45RA^+^CCR7^+^), central memory T cell (CD45RA^−^CCR7^+^), effector memory T cell (T_EM_) (CD45RA^−^CCR7^−^), and T_EMRA_ (CD45RA^+^CCR7^−^) phenotypes. A representative dot plot of the gating strategy used is shown (top left). The bar chart shows the relative CD6 surface expression on the indicated subsets from five different donors (top right). Data represent the percent (mean ± SEM) increase/reduction of Geo MFI values for each subset considering 100% the values for naïve T cells. *p < 0.05; **p < 0.01; ***p < 0.001 (unpaired *t* test). Lower panel, CD6 surface levels on late (CD27^lo/−^) and early (CD27^hi^) CD4^+^ T_EM_ (left), and T_EMRA_ (right) cells. AU, autofluorescence. **(C)** Same as in **(B)** but for CD8^+^ peripheral T cells.

Demonstration of CD6 down-modulation occurrence *ex vivo* was obtained by analyzing CD6 expression on the small population of peripheral CD25^+^ T cells from healthy donors. The results showed significantly lower CD6 surface levels on both CD4^+^CD25^+^ and CD8^+^CD25^+^ cells compared to their CD25^−^ counterparts (Figure 3A), according to our *in vitro* data. Similar studies were then extended to other peripheral T-cell populations related to signal strength and/or extent during T-cell activation events, namely central [central memory T cell (T_CM_)] and effector [effector memory T cell (T_EM_), T_EMRA_] memory cells (30, 31). As shown in Figures 3B,C, significant lower CD6 surface expression levels were observed in T_EM_ and T_EMRA_ cells from both CD4^+^ and CD8^+^ compartments compared to naïve and T_CM_ cells. Within T_EM_ and T_EMRA_ populations, the lowest CD6 surface levels were observed among the late subset, which expresses low/negative levels of CD27 (CD27^lo/−^) (Figures 3B,C), a co-stimulatory molecule of the TNFR family involved in the maintenance of proliferative capabilities of activated T cells and the generation of T-cell memory (32). Taken together, these results support that CD6 downregulation also occurs *in vivo* in the context of activation-related phenomena and effector memory T-cell generation.

### CD6 Down-Modulation Is Dependent on Intracellular Ca^2+^ Influx

**Figure 4:**
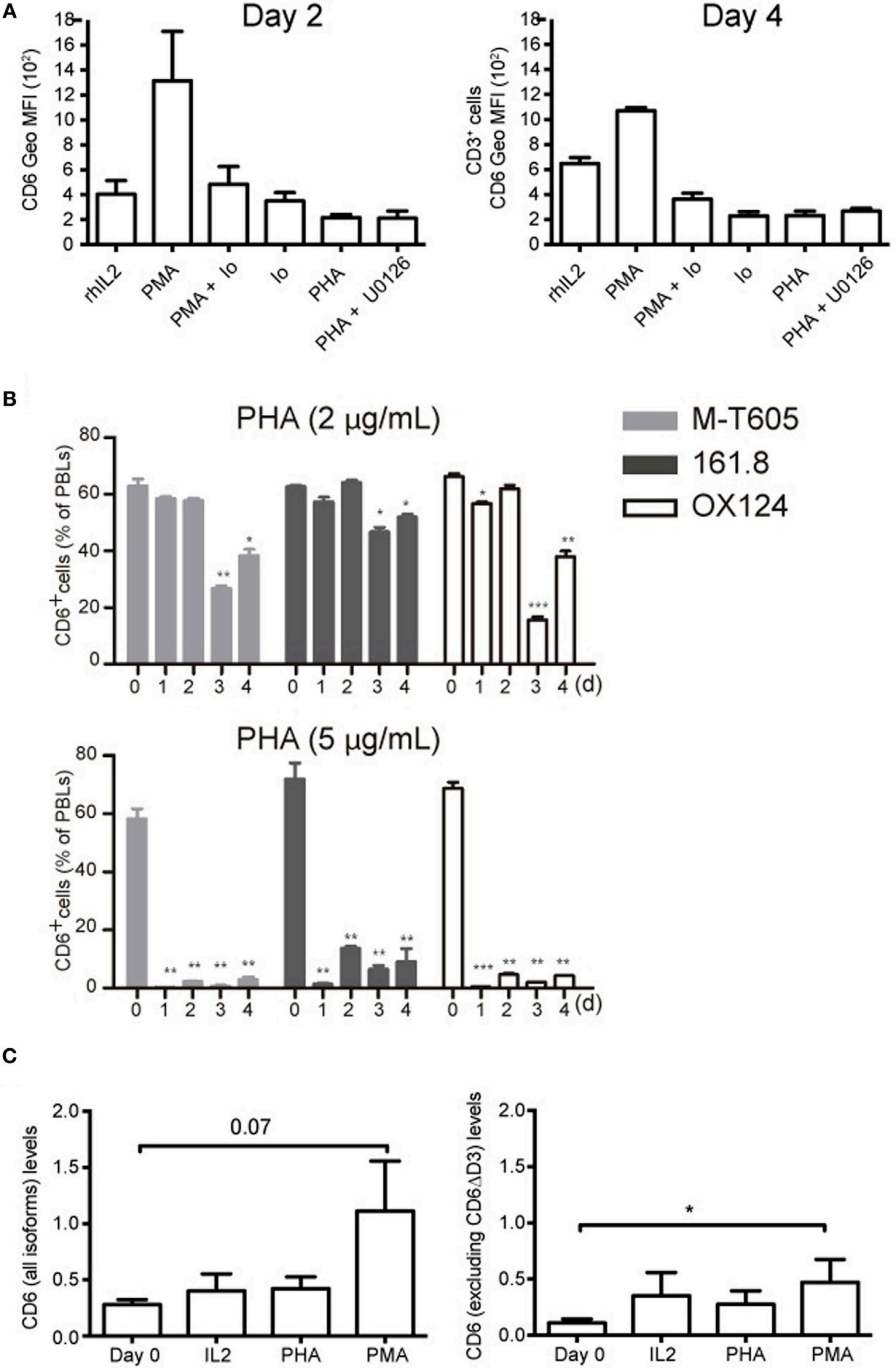
CD6 down-modulation is dependent on Ca^2+^ influx and does not involve differential utilization of extracellular CD6 isoforms nor changes in CD6 mRNA levels. **(A)** PBL (2 × 10^5^/well) was cultured for 2 (left) or 4 (right) days in the presence of rhIL-2 (10 ng/mL) alone, or together with PMA (10 ng/mL), PMA (10 ng/mL) plus Ionomycin (0.7 μg/mL), Ionomycin (0.7 μg/mL), PHA (5 μg/mL), and PHA plus U0126 (10 μM). Cells were stained with labeled mAb for CD3 (UCTH1) and CD6 (M-T605) and analyzed by flow cytometry. Shown is a bar chart representing the mean ± SD of geometric mean of fluorescence intensity (Geo MFI) values for CD6 expression. **(B)** PBLs (10^5^/well) cultured for 3 days in the presence of 2 μg/mL (top panel) or 5 μg/mL (bottom panel) of PHA were analyzed by flow cytometry for CD6 surface expression with either FITC-labeled M-T605 mAb or unlabeled OX124 and 161.8 mAb plus FITC-labeled goat anti-mouse Ig. Bars represent the percent (mean ± SEM) of CD6^+^ PBL at different time points following exposure to PHA, from three independent experiments performed. Comparisons were made with values at day 0. *p < 0.05; **p < 0.01; ***p < 0.001 (unpaired *t* test). **(C)** Reverse transcription PCR analysis of CD6 mRNA levels following 24 h stimulation of PBMC with PHA (5 μg/mL), PMA (10 ng/mL), or rhIL-2 (10 ng/mL). Left, results obtained with an exon 1–2 boundary-specific probe. Right, results obtained with an exon 5–6 boundary specific. Data are presented as mean ± SD (*n* = 4). *p < 0.05 (Student’s *t*-test).

To get some mechanistic insight into CD6 down-modulation, the effect of different pharmacological agents was explored. As shown in Figure 4A, ionomycin (0.7 μg/mL) either alone or in combination with PMA (10 ng/mL) down-modulated surface CD6 expression to a similar extent than induced by PHA (5 μg/mL), indicating that Ca^2+^ influx-dependent events are involved in CD6 down-modulation. These data, together with the fact that PMA stimulation increased CD6 expression, implies that PKC does not play a relevant role in CD6 downregulation. Finally, the selective inhibitor of MAP kinase kinases (MEK1/2) U0126 was unable to reverse the effects of PHA stimulation (Figure 4A), arguing also against a relevant contribution of the MAPK pathway in the down-modulation of surface CD6 expression.

### CD6 Down-Modulation Does Not Involve Changes in CD6 Isoform Utilization or mRNA Levels

T-cell activation has been reported to upregulate the expression of an alternatively spliced CD6 extracellular isoform (CD6ΔD3) lacking the CD166/ALCAM-binding domain (D3) (33). To explore this possibility as a temptative cause for CD6 down-modulation, PBLs were stained with different anti-CD6 mAbs at different time points post-PHA stimulation. As illustrated in Figure 4B (Figure S2 in Supplementary Material), the downregulated CD6 expression was detectable by the 161.8 mAb (14) but also the non-overlapping M-T605 (recognizing the amino-terminal D1 domain) (11) and OX124 (recognizing the CD166/ALCAM-binding D3 domain) mAbs (10) in a time- and dose-depending manner. This would indicate that CD6 down-modulation involves reduced surface expression of full-length and/or D3-deficient (CD6ΔD3) isoforms.

Next, putative changes in CD6 mRNA expression levels following T-cell activation were further assessed by RT-qPCR using probes specific for the exon 1–2 boundary (present in all reported CD6 isoforms) or the exon 5–6 boundary (absent from CD6ΔD3 isoforms). As illustrated in Figure 4C, PMA increased the levels of CD6 mRNA while both PHA and rhIL-2 did not induce significant changes. This would argue against reduced CD6 transcription as a cause of surface CD6 down-modulation following T-cell activation.

### sCD6 Is Released following T-Cell Activation

**Figure 5:**
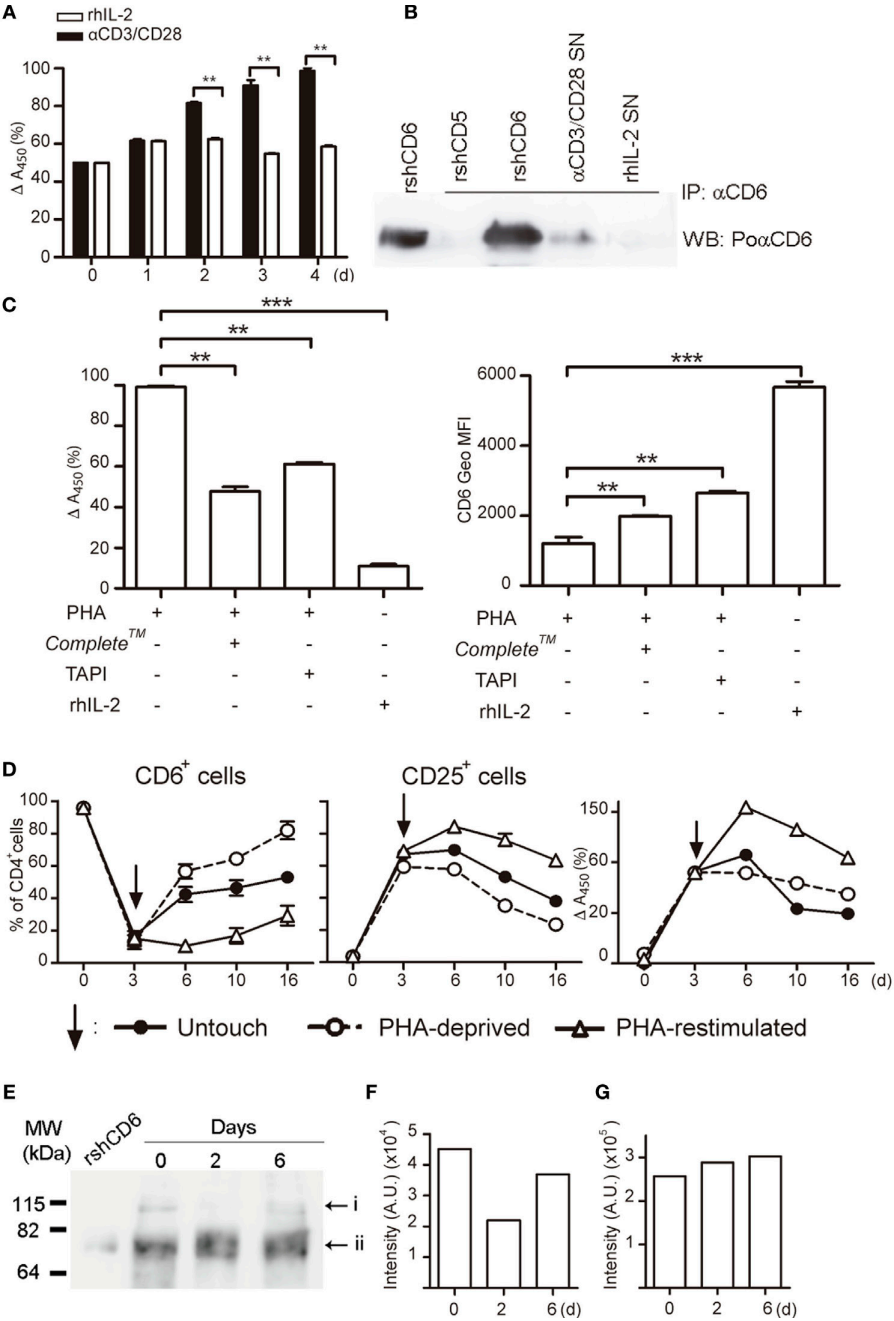
Activation-induced CD6 down-modulation involves shedding by proteolytic ectoenzymes, and CD6 surface expression recovers after the removal of activating stimuli. **(A)** Supernatants (SN) (100 μL) from PBLs (10^5^/well) cultured in the presence of αCD3/CD28 (2 μL) or rhIL-2 (10 ng/mL) were sampled daily for soluble CD6 (sCD6) release by ELISA. Data are expressed as percent (mean ± SEM) of *A*_450 nm_ values from three experiments performed taking as 100% the values obtained from 4-day αCD3/CD28-stimulated samples. **(B)** SN from PBLs (1–2 × 10^6^/well) cultured for 4 days as in **(A)** was immunoprecipitated with 161.8 mAb coupled to protein G sepharose beads. Recombinant soluble human CD6 (rshCD6) and rshCD5 (500 ng each) were included as positive and negative immunoprecipitation controls. Shown are chemiluminiscent results of Western blotting immunoprecipitates with rabbit anti-CD6 antiserum plus HRP-labeled donkey anti-rabbit Igs. Purified rshCD6 (500 ng) was included as a positive control for Western blot. **(C)** PHA-stimulated (2 μg/mL) PBL were cultured for 4 days in the presence or absence of TAPI-2 (100 mM), Complete™, or rhIL-2 (10 ng/mL). Left, bar chart showing the ELISA analysis of sCD6 in culture supernatants as done in **(A)**. Right, bar chart showing the geometric mean of fluorescence intensity (Geo MFI) (mean ± SEM) values for CD6 surface expression from three experiments performed. Comparisons were made with cells cultured with rhIL-2. **p < 0.01; ***p < 0.001 (unpaired *t* test). **(D)** PHA-stimulated (1 μg/mL) PBLs (10^5^/well) for 3 days (arrow) were either stimulus deprived by extensive washing (open circles), re-stimulated with PHA (open triangles), or left untouched (closed circles) for additional days of culture. Results represent the percent (mean ± SEM) of CD6^+^ (left) and CD25^+^ (middle) cells in the CD4^+^ subset from three different experiments. The right hand line chart shows sCD6 levels measured as in **(A)** and expressed as percent increase/decrease of *A*_450 nm_ values considering 100% the values obtained from 3-day PHA-stimulated samples. **(E,F)** Whole cell extracts from αCD3/CD28-stimulted PBMC for 0–6 days were western blotted for anti-CD6 reactivity. Shown is the chemiluminiscent results **(E)** together with densitometry analysis of i **(F)** and ii **(G)** bands with ImageJ software (AU, arbitrary units). The experiment shown is representative of four performed from different PBMC donors.

Next, it was explored whether the decreased surface CD6 expression could result from proteolytic cleavage of the membrane-bound form as previously reported for CD5 (34). The ELISA analysis of culture SN from PBLs stimulated with αCD3/CD28 (Figure 5A) or PHA (Figures 5C,D) showed significant increased levels of sCD6 from day 2 compared with cells exposed to rhIL-2. Western blot analysis of CD6 immunoprecipitates from αCD3/CD28-activated cell SN rendered a CD6-reactive band with similar molecular mass (~70 kDa) to rshCD6 used as a positive control (Figure 5B). Reactivity was not observed in immunoprecipitates from rhIL-2 treated SN or from recombinant rhsCD5 (Figure 5B). The addition of a protease inhibitor cocktail (Complete™) significantly reduced, though incompletely, both the release of sCD6 and the surface CD6 down-modulation (Figure 5C) from activated PBLs. Similar results were obtained using TAPI-2 (Figure 5C), a broad metalloproteinase inhibitor (35) with relative specificity for TNF-α-converting enzyme (TACE; also named ADAM17), an enzyme involved in the shedding of several surface proteins including the CD6 ligand (CD166/ALCAM) (36). No differences in cell viability were detected at the end of the stimulation period among all the samples analyzed (data not shown). Altogether, these results suggest that activation-induced surface CD6 down-modulation may result, at least in part, from proteolytic release involving members of the ADAM (a disintegrin and metalloproteinase) family.

### CD6 Surface Expression Recovers after Stimulation Deprival

The putative reversibility of PHA-induced CD6 downregulation on CD4^+^ T cell was next investigated by analyzing CD6 and CD25 expressions upon re-stimulation with PHA, PHA deprivation (by extensive washing), or left untouched for an additional period of time (Figure 5D). PHA-deprived cells gradually recovered CD6 expression over time, while it stayed low for PHA re-stimulated cells. In a similar manner, CD25 expression level gradually decreased once the cells were PHA deprived or left untouched, while it remained high upon PHA re-stimulation. Accordingly, sCD6 levels in culture SNs slowly declined unless cells were re-stimulated (Figure 5D, right hand side). This indicates that CD6 downregulation reverses *in vitro* if cells are no longer actively stimulated.

Western blot analysis of whole cell extracts from PBLs stimulated or not with αCD3/CD28 revealed two CD6-reactive bands of ~115 and ~75 kDa (Figures 5E–G), likely corresponding to fully processed membrane-bound and unprocessed intracellular forms, respectively (37). While the intensity of the ~75 kDa band remained mostly unchanged over time, there was a drop in the intensity of the ~115 kDa band at day 2 post-αCD3/CD28 stimulation that recovered by day 6 (Figures 5E–G). This result mimics the above reported kinetic analysis of surface CD6 expression (Figure 5D) and supports the assumption that the ~115 kDa band corresponds to membrane-bound CD6.

### CD6 Downregulation Correlates with Increased Susceptibility to Apoptosis

**Figure 6:**
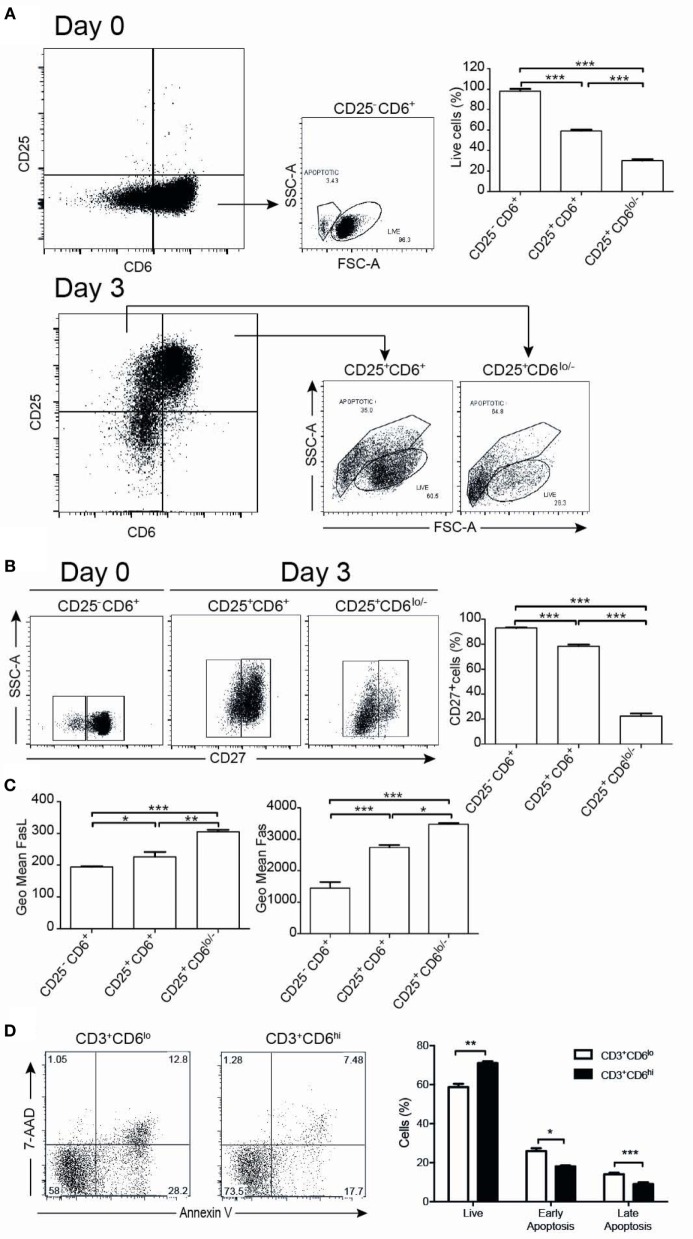
CD6 surface expression inversely correlates with T-cell viability. PBL (10^5^/well) exposed to αCD3/CD28 for 3 days was analyzed at days 0 and 3 by flow cytometry for cell viability and the expression of survival/apoptosis-related surface markers (CD3, CD25, CD6, CD27, CD95, and CD178). **(A)** Representative dot plots of live and apoptotic cells from day 0 (unstimulated) CD25^−^CD6^+^ cells (top panels) and day 3 αCD3/CD28-activated CD25^+^CD6^+^ and CD25^+^CD6^lo/−^ cells (bottom panels) as determined by FSC and SSC criteria. At the top right hand side, a bar chart is shown representing the percent of live cells in the indicated unstimulated (day 0, CD25^−^CD6^+^) and activated (day 3, CD25^+^CD6^+^ and CD25^+^CD6^lo/−^) T cells. **(B)** Representative dot plot analysis of CD27 expression versus SSC in the same days 0 and 3 cells as in **(A)**. The bar chart represents the percent of CD27^+^ cells in CD25^−^CD6^+^ cells (day 0) and CD25^+^CD6^+^ and CD25^+^CD6^lo/−^ cells (day 3). **(C)** Bar chart represents geometric mean of fluorescence intensity (mean ± SD of triplicates) of FasL (left) and Fas (right) expressions from same subsets as in **(A)** and are representative of two experiments. *p < 0.05; **p < 0.01; *** *p* < 0.001 (unpaired ANOVA test followed by Tukey’s multiple comparison test). **(D)** PBL (2 × 10^5^/well) were cultured for 24 h in the presence of rhIL-2 (10 ng/mL) plus PMA (10 ng/mL) and stained with labeled mAb for CD3 (MEM-57-APC), CD6 (M-T605-FITC), 7-AAD, and Annexin-PE. Left, representative dot plots of gated CD3^+^CD6^lo/–^ and CD3^+^CD6^hi^ cells post-PMA stimulation stained for Annexin V-PE and 7-AAD. Right, bar chart showing the percent (mean ± SEM) of live (Annexin-V^–^ 7-AAD^–^), early apoptotic (Annexin-V^+^ 7-AAD^–^), and late apoptotic (Annexin-V^+^ 7-AAD^+^) cells from PMA-stimulated CD3^+^CD6^lo/−^ and CD3^+^CD6^hi^ cells. *p < 0.05; **p < 0.01; ***p < 0.001 (Student’s *t*-test).

The functional significance of CD6 down-modulation upon T-cell activation was further explored by analyzing the number of live/apoptotic cells, as well as the expression of surface markers from the TNF superfamily (CD27, CD95/Fas, and CD178/FasL) on CD6^+^ versus CD6^lo/−^ activated T cells (CD3^+^CD25^+^). As illustrated by Figure 6A, 3-day αCD3/CD28 stimulation resulted in the appearance of CD25^+^CD6^lo/−^ cells showing significant lower levels of live cells compared to CD25^+^CD6^+^ or unstimulated CD25^−^CD6^+^ cells (day 0), as determined by forward and side scatter analyses. Moreover, compared with CD25^+^CD6^+^ cells, CD25^+^CD6^lo/−^ cells expressed significant lower surface levels of CD27, a co-stimulatory molecule (38) involved in resistance to apoptosis (Figure 6B). In contrast, the CD25^+^CD6^lo/−^ cells expressed significant higher surface levels of the AICD markers CD95/Fas and CD178/FasL than CD25^+^CD6^+^ cells (Figure 6C). These results suggest that reduction of CD6 surface levels correlates with a reduced T-cell viability and with markers of increased sensibility to apoptosis.

As PMA-induced activation upregulates CD6 expression, we also compared the apoptosis levels among PMA-activated T cells expressing different CD6 surface levels. The analysis of cell viability by annexin V and 7-AAD criteria indeed showed a higher frequency of viable cells among PMA-stimulated PBMC with higher CD6 surface levels compared with those expressing lower ones (Figure 6D). Thus, higher CD6 upregulation also correlates with higher viability among PMA-stimulated cells.

### Sorted CD25^+^CD6^hi^ and CD25^+^CD6^lo/−^ T Cells Differ in Their Proliferative Responses

**Figure 7:**
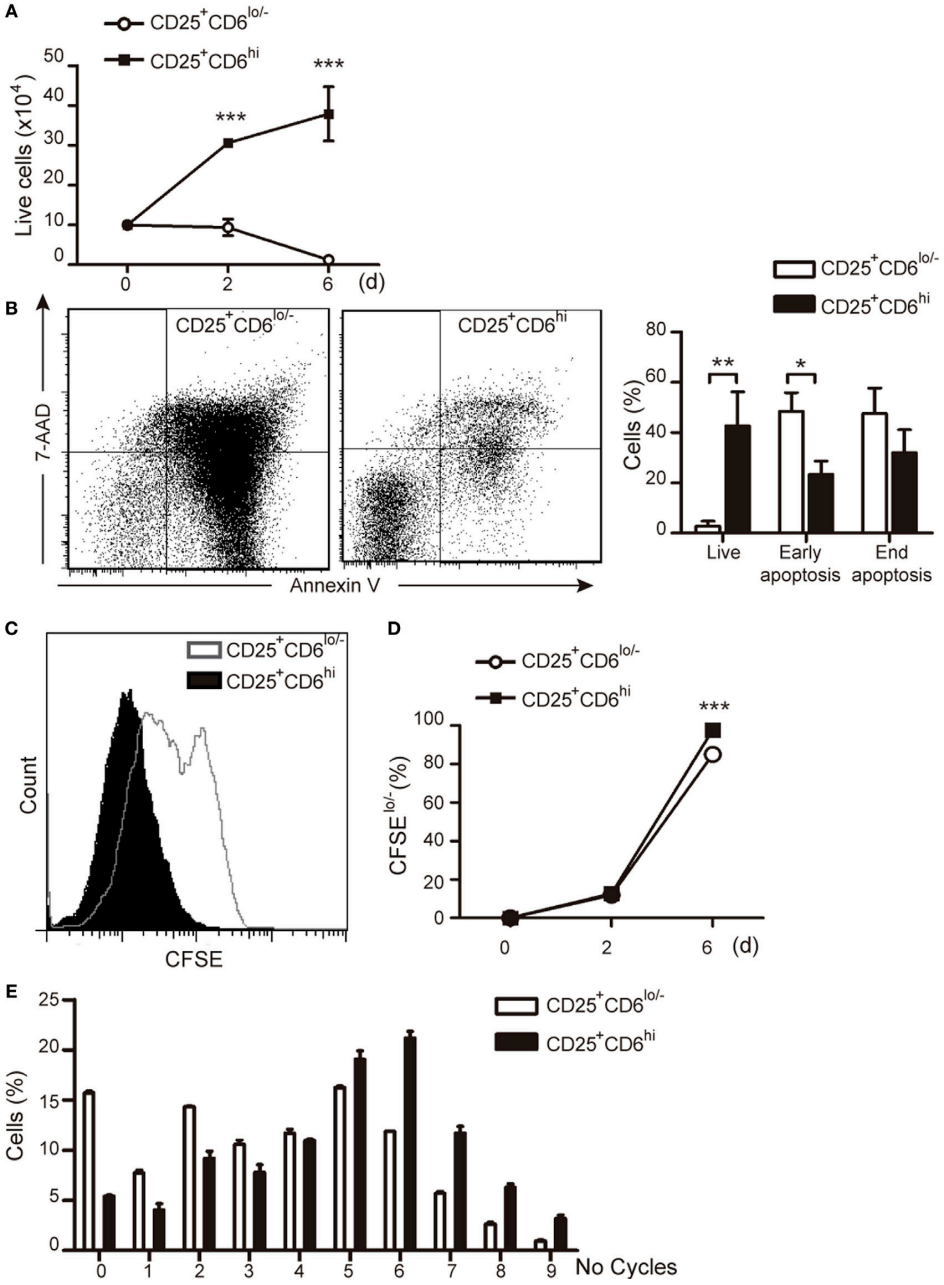
CD6 surface levels correlate T-cell viability and proliferative capability. PBLs (5 × 10^7^) exposed to PHA (2 μg/mL) for 3 days were subjected to cell sorting for isolation of CD3^+^CD25^+^ CD6^lo/−^ and CD6^hi^ subsets. Sorted cells were plated (10^5^/well) either with media alone **(A,B)** or stained with CFSE and co-cultured with allogeneic staphylococcal enterotoxin B (SEB)-loaded mature DC (mDC) (2 × 10^3^) **(C–E)** for 6 days. **(A)** Number of total live cells (measured by trypan blue dye exclusion) at different time points are represented. **(B)** Left, representative dot plots of Annexin-V-FITC and 7-AAD staining of CD25^+^CD6^lo/−^ and CD25^+^CD6^hi^ cells at day 6 post-sorting. Right, bar chart representing the percent of live cells (Annexin-V^−^ 7-AAD^−^), end apoptotic (Annexin-V^+^ 7-AAD^+^) and early apoptotic (Annexin-V^+^ 7-AAD^−^) cells. **(C)** Representative histogram showing CFSE staining of sorted CD25^+^CD6^hi^ and CD25^+^CD6^lo/−^ T cells co-cultured with SEB-loaded mDC for 6 days. **(D)** Percentage of proliferating (CFSE^lo/−^) CD25^+^CD6^hi^ and CD25^+^CD6^lo/−^ cells at days 2 and 6 post co-culture with allogeneic SEB-pulsed mDC is shown. Data are mean ± SD of triplicates from a representative experiment of two performed. *p < 0.05; **p < 0.01; ***p < 0.001 (unpaired *t* test).

In another set of experiments, the functional capabilities of sorted PHA-activated CD25^+^CD6^lo/−^ and CD25^+^CD6^hi^ T cells (Figure S3 in Supplementary Material) were further explored by culturing them for an additional 6-day period and assessing their viability and proliferative responses. As illustrated in Figure 7A, the number of viable CD25^+^CD6^lo/−^ cells decreased over time when they were left unstimulated, while that of CD25^+^CD6^hi^ significantly increased during the same period of time, as measured by trypan blue dye exclusion. Moreover, FACS analysis of the same cells co-stained with Annexin-V-FITC and 7-AAD (Figure 7B) showed both a significant reduction of live cells and an increase in early apoptotic cells within the CD25^+^CD6^lo/−^ subset compared to CD25^+^CD6^hi^ cells.

The proliferative responses of sorted PHA-activated CD25^+^CD6^hi^ and CD25^+^CD6^lo/−^ T cells were also investigated by co-culture with SEB-loaded allogeneic mDCs. As illustrated in Figures 7C–E, CD25^+^CD6^lo/−^ T cells showed a significant lower percentage of CFSE^lo/−^ cells as compared to their CD25^+^CD6^hi^ counterparts. Taken together, these results further support that CD6 downregulation correlates with apoptosis and reduced proliferative capabilities of T cells.

### Modulation of CD6 Expression Influences the Survival of Leukemic T Cells

**Figure 8:**
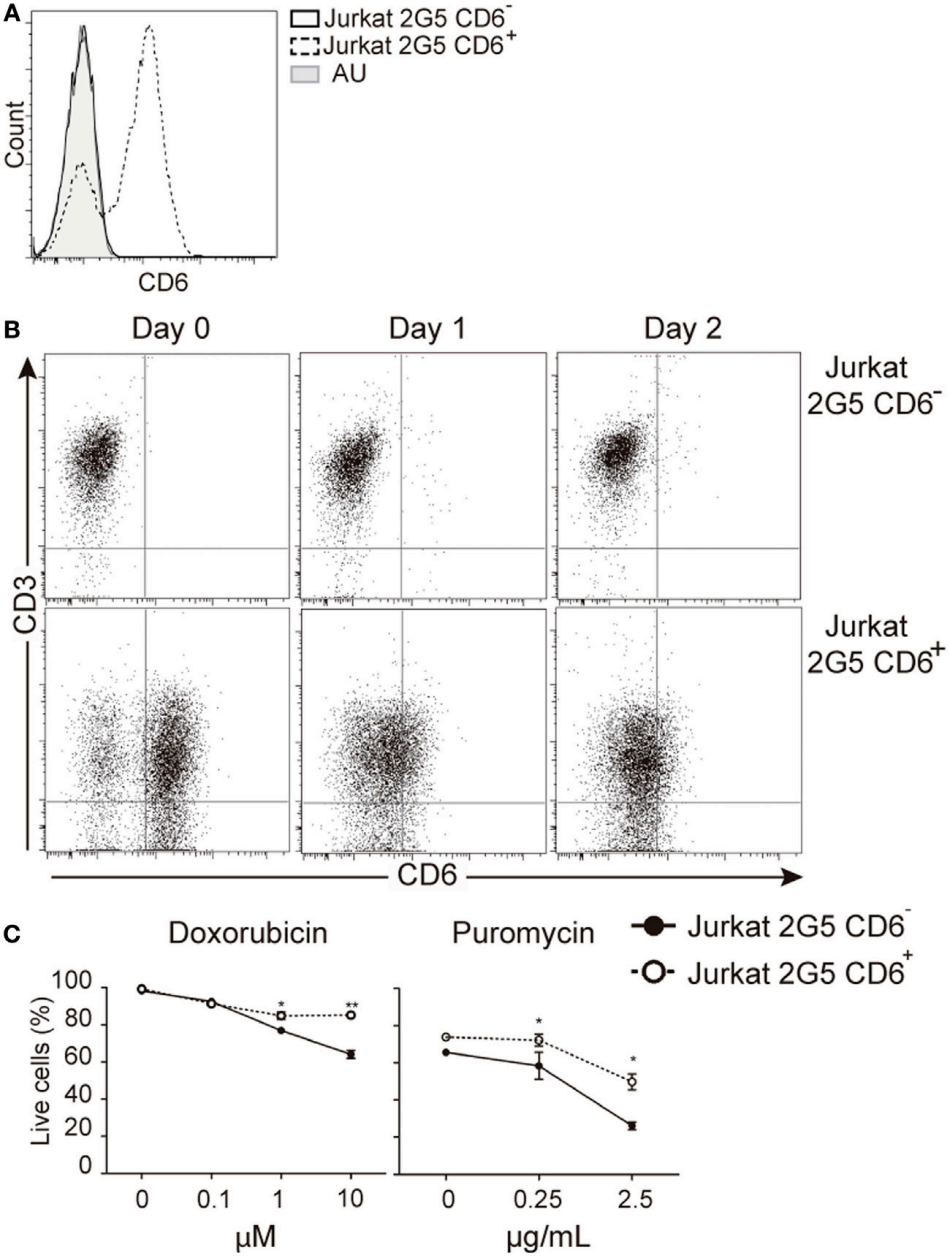
CD6 surface expression attenuates T-cell receptor-independent induced apoptosis in leukemic cells. **(A)** CD6 expression in parental (CD6^−^) and stably CD6-transfected (CD6^+^) 2G5 Jurkat T cells. **(B)** Representative dot plots showing CD6 and CD3 expressions in parental (top) and CD6-transfected (bottom) 2G5 cells before (day 0) and after (day 1 and 2) PHA (2 μg/mL) stimulation. **(C)** Percent of live cells (Annexin-V^−^ 7-AAD^−^) in CD6^−^ and CD6^+^ 2G5 cells exposed to increasing concentrations of doxorubicin or puromycin for 1 day. Results represent mean ± SD of triplicates from one representative experiment of two performed. *p < 0.05; **p < 0.01; ***p < 0.001 (unpaired ANOVA test followed by Bonferroni posttest).

The protective effect of CD6 expression on TCR-induced apoptosis would agree with previous reports showing that CD6 ligation prevents IgM cross-linking-induced apoptosis of leukemic B cells (chronic lymphocytic leukemia) (39). It was then investigated whether surface CD6 expression influences apoptosis induced by genotoxic agents overcoming the need of signaling *via* TCR. To that end, we used untransfected and CD6-transfected 2G5 cells, a CD6-deficient Jurkat T-cell derivative (14, 26) (Figure 8A). As shown in Figure 8B, 2G5 cell transfectants underwent down-modulation of CD6 surface expression by days 1 and 2 post-PHA activation, in a similar manner to that observed for peripheral T cells. Furthermore, CD6-transfected 2G5 cells were consistently more resistant to apoptosis induced by doxorubicin (0.1–10 μM) or puromycin (0.25–2.5 μg/mL) than parental (untransfected) cells (Figure 8C). These data support the notion that CD6 rescues lymphocytes from apoptosis induced also by TCR-independent pathways.

## Discussion

The current view is that CD6—through its interaction with CD166/ALCAM—is involved in positive selection and survival of developing mouse thymocytes (18, 40), as well as in early adhesive APC–T-cell contacts driving to formation of mature IS and further proliferative responses of human T cells (9–11). In the present work, evidence is provided on the involvement of CD6 down-modulation in the regulation of late human T-cell activation events, rendering cells hyper-sensitive to apoptosis and hypo-responsive to further proliferative stimulation.

Surface downregulation of CD6 was observed on resting human T cells upon *in vitro* activation with different TCR-dependent stimuli (PHA, αCD3/CD28, and SEB-loaded allogeneic mDC), which in turn upregulated CD25 expression. These activation-induced T-cell changes were reversible since expression of both CD6 and CD25 recovered upon stimulus removal. Interestingly, CD6 downregulation was observed in CD25^+^ T cells independently of FoxP3 co-expression. Consequently, CD6 was found downregulated on T cells with both activated and regulatory phenotypes, thus arguing against being a characteristic exclusive of human T_reg_ as recently proposed (22). Thus, CD6 downregulation would depend on T-cell activation signals irrespective of the differentiation pathway (effector or regulatory) that the cell undergoes.

The *in vivo* occurrence of a small proportion of circulating T cells from healthy individuals with downregulated CD6 surface expression and diminished alloreactivity was reported some time ago (21), although their origin could not be determined. The *ex vivo* analysis presented here demonstrates the expression of lower CD6 surface levels on CD25^+^ T cells from healthy donors compared to their CD25^−^ counterparts. In addition, T_EM_ and T_EMRA_ subsets from both the CD4^+^ and CD8^+^ compartments expressed significantly reduced CD6 surface levels compared to naïve and T_CM_-cell populations. These data indicate that CD6 downregulation also occurs *in vivo* in the context of activation-related phenomena such as generation of effector memory cells (T_EM_ and T_EMRA_) and are in agreement with a signal strength/duration-dependent and progressive model by which a naïve T-cell progresses to T_CM_ and then to T_EM_/T_EMRA_ (31, 41).

Different mechanisms by which CD6 functional capabilities would be severely afflicted upon lymphocyte activation-related events have already been reported. Mitogen-activated PBL is known to express no less than five different alternatively spliced CD6 cytoplamic isoforms involving loss of structural motifs relevant to signal-transducing function (42, 43). Likewise, the extracellular isoform (CD6Δ3) lacking the CD166/ALCAM-binding domain (D3) of CD6—and consequently unable to localize at the APC–T-cell interface during IS formation—is upregulated and dominates upon T-cell activation (33). The present report shows shedding of membrane-bound CD6 as an additional mechanism previously suspected but unproven (24, 25). However, we cannot exclude other alternative and/or complementary mechanism contributing to CD6 down-modulation, as it is the case of internalization *via* coated pits or c-Cbl-mediated ubiquitination previously reported for CD5 (44–46). The surface receptor levels are often regulated by endocytosis and subsequent lysosomal degradation or recycling to plasma membrane, depending on whether they are ubiquitinated or not. The Cbl family of ubiquitin ligases is highly expressed in T cells and deeply involved in negative regulation of T-cell activation. Available evidence points to the role of Cbl ligases in regulating the surface levels of CD5 (44, 47), and this could also apply to the sister molecule CD6. Thus, T-cell activation could contribute to reduced CD6 surface levels by both proteolytic events and Cbl-mediated ubiquitination, which could be direct or indirect through its physical association with CD5. Regarding the putative contribution of alternative splicing to membrane-bound CD6 down-modulation, available reports by independent groups (33, 42, 43) have not identified alternatively spliced mRNAs coding for sCD6 and/or CD6 isoforms devoid of its entire extracellular region.

The present data indicate that CD6 shedding is due to proteolytic cleavage by, at least in part, members of the ADAM family of metalloproteinases. TACE/ADAM17 is known to generate soluble forms of several lymphocyte surface receptors (e.g., CD62L, CD16, CD166/ALCAM, ICOS ligand, and FasL) in the context of cell activation (36, 48–51) and would be a feasible candidate. However, the fact that CD6 down-modulation was induced by ionomycin but not by PMA (Figure 4A) would favor the putative involvement of ADAM10, as reported for other transmembrane receptors (52).

The functional role of sCD6 release in T-cell physiology is uncertain. Available *in vitro* evidence shows that sCD6 interferes with T-cell activation and IS maturation (1, 9, 10). Based on this, it can be speculated that sCD6 would act as a “decoy receptor” contributing to the inactivation of bystander T cells at inflammation sites. In any case, the data collectively point to the existence of different levels of the regulation of human CD6 function during late T-cell activation, all of them involving physical loss of relevant extracellular and/or intracytoplasmic regions.

Interestingly, CD6 downregulation was not observed upon *in vitro* activation of mouse T cells or *ex vivo* analysis of spleen CD25^+^ (either FoxP3^+^ or FoxP3^−^) T cells (Figures S4A,B in Supplementary Material), thus stressing the existence of inter-species differences. Nevertheless, the modulation of mouse CD6 function by mechanisms other than surface downregulation (e.g., expression of alternatively spliced extracellular and/or intracytoplasmic isoforms) is still possible and would deserve further investigation.

The functional consequences of human CD6 downregulation reported here are (i) impaired T-cell proliferative capabilities and (ii) higher susceptibility to apoptosis (namely AICD), as deduced from the analysis of *in vitro*-generated and sorted CD25^+^CD6^lo/−^ and CD25^+^CD6^hi^ cells. The former is illustrated by the lower numbers of cycling CD25^+^CD6^lo/−^ lymphocytes observed upon stimulation with SEB-loaded allogeneic mDC, as compared to their CD25^+^CD6^hi^ counterparts. This agrees with the critical role assigned to CD6–CD166/ALCAM interaction for IS formation and maturation (9–11) as well as for TCR signaling (17). The higher levels of apoptotic cells and of AICD markers (CD95/Fas and CD178/FasL) in CD25^+^CD6^low/−^ T cells also agree with the anti-apoptotic properties assigned to CD6 (39). Ligation of CD6 on T cells by specific mAbs or CD166/ALCAM activates the MAPK cascade (14), which is known to result in higher levels of mcl-1 and promotion of cell survival (53). A similar mechanism would apply for the modulation of bcl-2/Bax equilibrium observed in B cells following IgM-induced apoptosis (39). The positive effect of CD6 in T-cell survival and proliferation would not be restricted to humans as CD6-deficient mouse T cells also exhibit a limited proliferation and increased apoptosis following *in vitro* activation (54). The intriguing fact is that the anti-apoptotic effect of CD6 is not necessarily linked to TCR cross-linking, since it is also evidenced upon corticosteroid (39), Galectin-1 and -3 (27), and as shown here, genotoxic-induced apoptosis, which all by-pass such a relevant lymphocyte receptor complex. Indeed, the sole presence of CD6 was sufficient to reduce the deleterious effects of doxorubicin or puromycin in leukemic Jurkat T cells. These results not only confirm the TCR-independent anti-apoptotic properties of CD6 but also open the possibility of sensitizing CD6-positive leukemic T cells to increase the efficacy and/or reduce the toxicity of chemotherapeutic agents as recently proposed (55).

Altogether, the present report provides new insight into the origin of sCD6 and the circulating CD6^−^ T-cell subset reported long ago (21, 24), as well as into the functional consequences of CD6 down-modulation on ongoing T-lymphocyte responses. The observed effects on the attenuation of T-cell proliferative responses and sensitization to apoptotic events place CD6 as a new player in T-cell homeostatic processes.

## Ethics Statement

This study was carried out in accordance with the recommendations and approval by the Comité Ético de Investigación Clínica (CEIC) from the Hospital Clínic of Barcelona.

## Author Contributions

ECarrasco designed and performed the experiments, analyzed the data, and wrote and edited the manuscript. CE-F performed the experiments and edited the manuscript. CM-J, NC, IS-T, MM-F and AS participated in experimental design, and critically reviewed the manuscript. ECarreras designed and performed the experiments, analyzed the data, and edited the manuscript. FL supervised the research and wrote the manuscript.

## Conflict of Interest Statement

The authors declare that the research was conducted in the absence of any commercial or financial relationships that could be construed as a potential conflict of interest.

## Abbreviations

ADAM, a disintegrin and metalloproteinase; AICD, activation-induced cell death; ALCAM, activated leukocyte cell adhesion molecule; APC, antigen-presenting cell; CLL, chronic lymphocytic leukemia; Geo MFI, geometric mean of fluorescence intensity; mDC, mature dendritic cell; rshCD6, recombinant soluble human CD6; SRCR, scavenger receptor cysteine-rich; SEB, staphylococcal enterotoxin B; SN, supernatant; TACE, TNF-α-converting enzyme; T_CM_, central memory T cell; T_EM_, effector memory T cell; T_reg_, regulatory T cell.

## References

[1] Santos RF Oliveira L Carmo AM. Tuning T cell activation: the function of CD6 at the immunological synapse and in T cell responses. Curr Drug Targets (2016) 17(6):630–9. 10.2174/1389450116666150531152439 26028048

[2] Kofler DM Farkas A von Bergwelt-Baildon M Hafler DA. The link between CD6 and autoimmunity: genetic and cellular associations. Curr Drug Targets (2016) 17(6):651–65. 10.2174/1389450117666160201105934 26844569

[3] Hernández P Moreno E Aira LE Rodríguez PC. Therapeutic targeting of CD6 in autoimmune diseases: a review of Cuban clinical studies with the antibodies IOR-T1 and itolizumab. Curr Drug Targets (2016) 17(6):666–77. 10.2174/1389450117666160201114308 26844560

[4] Aruffo A Melnick MB Linsley PS Seed B The lymphocyte glycoprotein CD6 contains a repeated domain structure characteristic of a new family of cell surface and secreted proteins. J Exp Med (1991) 174:949–52. 10.1084/jem.174.4.949 1919444 PMC2118957

[5] Braun M Muller B ter Meer D Raffegerst S Simm B Wilde S The CD6 scavenger receptor is differentially expressed on a CD56 natural killer cell subpopulation and contributes to natural killer-derived cytokine and chemokine secretion. J Innate Immun (2011) 3:420–34. 10.1159/000322720 21178331

[6] Cortes F Deschaseaux F Uchida N Labastie MC Friera AM He D HCA, an immunoglobulin-like adhesion molecule present on the earliest human hematopoietic precursor cells, is also expressed by stromal cells in blood-forming tissues. Blood (1999) 93:826–37. 9920831

[7] Konno A Ahn JS Kitamura H Hamilton MJ Gebe JA Aruffo A Tissue distribution of CD6 and CD6 ligand in cattle: expression of the CD6 ligand (CD166) in the autonomic nervous system of cattle and the human. J Leukoc Biol (2001) 69:944–50. 11404380

[8] Robinson WH Neuman de Vegvar HE Prohaska SS Rhee JW Parnes JR Human CD6 possesses a large, alternatively spliced cytoplasmic domain. Eur J Immunol (1995) 25:2765–9. 10.1002/eji.1830251008 7589069

[9] Gimferrer I Calvo M Mittelbrunn M Farnos M Sarrias MR Enrich C Relevance of CD6-mediated interactions in T cell activation and proliferation. J Immunol (2004) 173:2262–70. 10.4049/jimmunol.173.4.2262 15294938

[10] Hassan NJ Barclay AN Brown MH Optimal T cell activation requires the engagement of CD6 and CD166. Eur J Immunol (2004) 34:930–40. 10.1002/eji.200424856 15048703

[11] Zimmerman AW Joosten B Torensma R Parnes JR van Leeuwen FN Figdor CG. Long-term engagement of CD6 and ALCAM is essential for T-cell proliferation induced by dendritic cells. Blood (2006) 107:3212–20. 10.1182/blood-2005-09-3881 16352806

[12] Bowen MA Patel DD Li X Modrell B Malacko AR Wang WC Cloning, mapping, and characterization of activated leukocyte-cell adhesion molecule (ALCAM), a CD6 ligand. J Exp Med (1995) 181:2213–20. 10.1084/jem.181.6.2213 7760007 PMC2192054

[13] Chappell PE Garner LI Yan J Metcalfe C Hatherley D Johnson S Structures of CD6 and its ligand CD166 give insight into their interaction. Structure (2015) 23:1426–36. 10.1016/j.str.2015.05.019 26146185 PMC4533223

[14] Ibanez A Sarrias MR Farnos M Gimferrer I Serra-Pages C Vives J Mitogen-activated protein kinase pathway activation by the CD6 lymphocyte surface receptor. J Immunol (2006) 177:1152–9. 10.4049/jimmunol.177.2.1152 16818773

[15] Gimferrer I Ibanez A Farnos M Sarrias MR Fenutria R Rosello S The lymphocyte receptor CD6 interacts with syntenin-1, a scaffolding protein containing PDZ domains. J Immunol (2005) 175:1406–14. 10.4049/jimmunol.175.3.1406 16034076

[16] Hassan NJ Simmonds SJ Clarkson NG Hanrahan S Puklavec MJ Bomb M CD6 regulates T-cell responses through activation-dependent recruitment of the positive regulator SLP-76. Mol Cell Biol (2006) 26:6727–38. 10.1128/MCB.00688-06 16914752 PMC1592849

[17] Roncagalli R Hauri S Fiore F Liang Y Chen Z Sansoni A Quantitative proteomics analysis of signalosome dynamics in primary T cells identifies the surface receptor CD6 as a Lat adaptor-independent TCR signaling hub. Nat Immunol (2014) 15:384–92. 10.1038/ni.2843 24584089 PMC4037560

[18] Singer NG Fox DA Haqqi TM Beretta L Endres JS Prohaska S CD6: expression during development, apoptosis and selection of human and mouse thymocytes. Int Immunol (2002) 14:585–97. 10.1093/intimm/dxf025 12039910

[19] Bott CM Doshi JB Li LL McMurtry SA Sanders JL Fox DA. Transcriptional regulation of CD6 expression on human T lymphocytes by phorbol ester. J Immunol (1994) 153:1–9. 8207228

[20] Borthwick NJ Bofill M Hassan I Panayiotidis P Janossy G Salmon M Factors that influence activated CD8+ T-cell apoptosis in patients with acute herpesvirus infections: loss of costimulatory molecules CD28, CD5 and CD6 but relative maintenance of Bax and Bcl-X expression. Immunology (1996) 88:508–15. 8881750 PMC1456646

[21] Rasmussen RA Counts SL Daley JF Schlossman SF. Isolation and characterization of CD6-T cells from peripheral blood. J Immunol (1994) 152:527–36. 7904289

[22] Garcia Santana CA Tung JW Gulnik S. Human Treg cells are characterized by low/negative CD6 expression. Cytometry A (2014) 85:901–8. 10.1002/cyto.a.22513 25088497

[23] Sarrias MR Farnos M Mota R Sanchez-Barbero F Ibanez A Gimferrer I CD6 binds to pathogen-associated molecular patterns and protects from LPS-induced septic shock. Proc Natl Acad Sci U S A (2007) 104:11724–9. 10.1073/pnas.0702815104 17601777 PMC1913855

[24] Ramos-Casals M Font J Garcia-Carrasco M Calvo J Places L Padilla O High circulating levels of soluble scavenger receptors (sCD5 and sCD6) in patients with primary Sjogren’s syndrome. Rheumatology (2001) 40:1056–9. 10.1093/rheumatology/40.9.1056 11561119

[25] Aibar J Martinez-Florensa M Castro P Carrasco E Escoda-Ferran C Fernandez S Pattern of soluble CD5 and CD6 lymphocyte receptors in critically ill patients with septic syndromes. J Crit Care (2015) 30:914–9. 10.1016/j.jcrc.2015.04.120 26031813

[26] Simarro M Calvo J Vila JM Places L Padilla O Alberola-Ila J Signaling through CD5 involves acidic sphingomyelinase, protein kinase C-zeta, mitogen-activated protein kinase kinase, and c-Jun NH2-terminal kinase. J Immunol (1999) 162:5149–55. 10227986

[27] Escoda-Ferran C Carrasco E Caballero-Banos M Miro-Julia C Martinez-Florensa M Consuegra-Fernandez M Modulation of CD6 function through interaction with Galectin-1 and -3. FEBS Lett (2014) 588:2805–13. 10.1016/j.febslet.2014.05.064 24945728

[28] Gimferrer I Farnos M Calvo M Mittelbrunn M Enrich C Sanchez-Madrid F The accessory molecules CD5 and CD6 associate on the membrane of lymphoid T cells. J Biol Chem (2003) 278:8564–71. 10.1074/jbc.M209591200 12473675

[29] Lozano F Alberola-Ila J Places L Gallart T Vives J. Protein kinase C-dependent up-regulation of CD5 surface expression on normal and lymphoblastoid T cells. Immunology (1990) 70:434–9. 1697562 PMC1384245

[30] Farber DL Yudanin NA Restifo NP. Human memory T cells: generation, compartmentalization and homeostasis. Nat Rev Immunol (2014) 14:24–35. 10.1038/nri3567 24336101 PMC4032067

[31] Sallusto F Geginat J Lanzavecchia A Central memory and effector memory T cell subsets: function, generation, and maintenance. Annu Rev Immunol (2004) 22:745–63. 10.1146/annurev.immunol.22.012703.104702 15032595

[32] Hendriks J Gravestein LA Tesselaar K van Lier RA Schumacher TN Borst J. CD27 is required for generation and long-term maintenance of T cell immunity. Nat Immunol (2000) 1:433–40. 10.1038/80877 11062504

[33] Castro MA Oliveira MI Nunes RJ Fabre S Barbosa R Peixoto A Extracellular isoforms of CD6 generated by alternative splicing regulate targeting of CD6 to the immunological synapse. J Immunol (2007) 178:4351–61. 10.4049/jimmunol.178.7.4351 17371992

[34] Calvo J Places L Espinosa G Padilla O Vila JM Villamor N Identification of a natural soluble form of human CD5. Tissue Antigens (1999) 54:128–37. 10.1034/j.1399-0039.1999.540203.x 10488739

[35] Crowe PD Walter BN Mohler KM Otten-Evans C Black RA Ware CF A metalloprotease inhibitor blocks shedding of the 80-kD TNF receptor and TNF processing in T lymphocytes. J Exp Med (1995) 181:1205–10. 10.1084/jem.181.3.1205 7869036 PMC2191902

[36] Gilsanz A Sanchez-Martin L Gutierrez-Lopez MD Ovalle S Machado-Pineda Y Reyes R ALCAM/CD166 adhesive function is regulated by the tetraspanin CD9. Cell Mol Life Sci (2013) 70:475–93. 10.1007/s00018-012-1132-0 23052204 PMC11113661

[37] Swack JA Mier JW Romain PL Hull SR Rudd CE. Biosynthesis and post-translational modification of CD6, a T cell signal-transducing molecule. J Biol Chem (1991) 266:7137–43. 2016320

[38] Croft M. Co-stimulatory members of the TNFR family: keys to effective T-cell immunity? Nat Rev Immunol (2003) 3:609–20. 10.1038/nri1148 12974476

[39] Osorio LM De SA Aguilar-Santelises M Mellstedt H Jondal M. CD6 ligation modulates the Bcl-2/Bax ratio and protects chronic lymphocytic leukemia B cells from apoptosis induced by anti-IgM. Blood (1997) 89:2833–41. 9108402

[40] Orta-Mascaró M Consuegra-Fernández M Carreras E Roncagalli R Carreras-Sureda A Alvarez P CD6 modulates thymocyte selection and peripheral T cell homeostasis. J Exp Med (2016) 213:1387–97. 10.1084/jem.20151785 27377588 PMC4986531

[41] Gasper DJ Tejera MM Suresh M. CD4 T-cell memory generation and maintenance. Crit Rev Immunol (2014) 34:121–46. 10.1615/CritRevImmunol.2014010373 24940912 PMC4062920

[42] Bowen MA Whitney GS Neubauer M Starling GC Palmer D Zhang J Structure and chromosomal location of the human CD6 gene: detection of five human CD6 isoforms. J Immunol (1997) 158:1149–56. 9013954

[43] Bonet L Farnos M Martinez-Florensa M Martinez VG Lozano F. Identification of functionally relevant phoshorylatable serine clusters in the cytoplasmic region of the human CD6 lymphocyte surface receptor. FEBS Lett (2013) 587:2205–13. 10.1016/j.febslet.2013.05.043 23711376

[44] Demydenko D. c-Cbl mediated ubiquitylation and regulation of cell surface exposure of CD5. Biochem Biophys Res Commun (2010) 392:500–4. 10.1016/j.bbrc.2010.01.052 20085752

[45] Lu X Axtell RC Collawn J Gibson A Justement LB Raman C. AP2 adaptor complex-dependent internalization of CD5: differential regulation in T and B cells. J Immunol (2002) 168:5612–20. 10.4049/jimmunol.168.11.5612 12023358

[46] Vila JM Calvo J Places L Padilla O Arman M Gimferrer I Role of two conserved cytoplasmic threonine residues (T410 and T412) in CD5 signaling. J Immunol (2001) 166:396–402. 10.4049/jimmunol.166.1.396 11123317

[47] Voisinne G Garcia-Blesa A Chaoui K Fiore F Bergot E Girard L Co-recruitment analysis of the CBL and CBLB signalosomes in primary T cells identifies CD5 as a key regulator of TCR-induced ubiquitylation. Mol Syst Biol (2016) 12:876. 10.15252/msb.20166837 27474268 PMC4965873

[48] Ebsen H Lettau M Kabelitz D Janssen O. Subcellular localization and activation of ADAM proteases in the context of FasL shedding in T lymphocytes. Mol Immunol (2015) 65:416–28. 10.1016/j.molimm.2015.02.008 25745808

[49] Marczynska J Ozga A Wlodarczyk A Majchrzak-Gorecka M Kulig P Banas M The role of metalloproteinase ADAM17 in regulating ICOS ligand-mediated humoral immune responses. J Immunol (2014) 193:2753–63. 10.4049/jimmunol.1302893 25108021

[50] Romee R Foley B Lenvik T Wang Y Zhang B Ankarlo D NK cell CD16 surface expression and function is regulated by a disintegrin and metalloprotease-17 (ADAM17). Blood (2013) 121:3599–608. 10.1182/blood-2012-04-425397 23487023 PMC3643761

[51] Wang Y Zhang AC Ni Z Herrera A Walcheck B. ADAM17 activity and other mechanisms of soluble l-selectin production during death receptor-induced leukocyte apoptosis. J Immunol (2010) 184:4447–54. 10.4049/jimmunol.0902925 20220092 PMC2858405

[52] Sanderson MP Erickson SN Gough PJ Garton KJ Wille PT Raines EW ADAM10 mediates ectodomain shedding of the betacellulin precursor activated by p-aminophenylmercuric acetate and extracellular calcium influx. J Biol Chem (2005) 280:1826–37. 10.1074/jbc.M408804200 15507448

[53] Le Gouill S Podar K Harousseau JL Anderson KC. Mcl-1 regulation and its role in multiple myeloma. Cell Cycle (2004) 3:1259–62. 10.4161/cc.3.10.1196 15467463

[54] Li Y Singer NG Whitbred J Bowen MA Fox DA Lin F. CD6 as a potential target for treating multiple sclerosis. Proc Natl Acad Sci U S A (2017) 114:2687–92. 10.1073/pnas.1615253114 28209777 PMC5347585

[55] Izquierdo Cano LS Espinosa Estrada EE Hernández Padrón C Ramón Rodríguez LG Ávila Cabrera OM Hernández Ramírez P Itolizumab humanized monoclonal antibody (anti-cd6) in patients with cd6+ lymphoproliferative disorders. Preliminary evidence. Rev Cubana Hematol Immunol Hemoter (2014) 30:257–64.

