# Supplementary material for: Human CD6 Down-Modulation following T-Cell Activation Compromises Lymphocyte Survival and Proliferative Responses
Source: Front Immunol. 2017 Jun 30;8:769. doi: 10.3389/fimmu.2017.00769 (PMC5492662; doi:10.3389/fimmu.2017.00769)
Supplement: Supplementary file 1 [file Data_Sheet_1.DOCX]

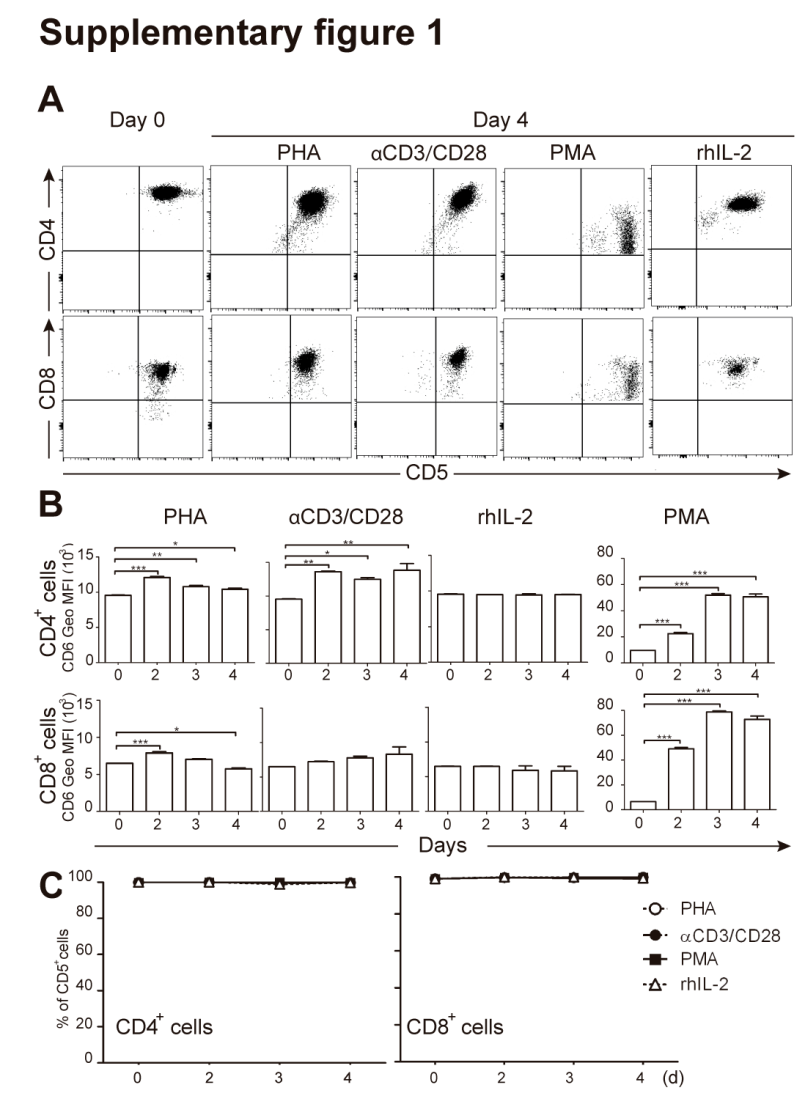


**Figure S1 Up-regulation of CD5 surface expression in response to different polyclonal T-cell activators**. PBLs (10^5^/well) cultured in the presence or absence of PHA (2 µg/mL), αCD3/CD28 beads (2 µL), PMA (10 ng/mL) or rhIL-2 (10 ng/mL) were analyzed at day 0 and 4 for CD5 surface expression (UCHT2) on CD4^+^ (MT310) and CD8^+^ (SK1) T-cell subsets. (**A**) Representative dot plots of CD5 expression on CD4^+^ (top panel) or CD8^+^ (bottom panel) cells at day 0 and 4 post PHA, αCD3/CD28, PMA or rhIL-2 exposure. (**B**) Bar charts showing Geo MFI (mean ± SD) of CD5 surface expression on CD4^+^ (top panel) or CD8^+^ (bottom panel) T-cells at different time points post PHA, αCD3/CD28, rhIL2 or PMA exposure. Data are from three independent experiments. Comparisons were made with values at day 0. *, p<0.05; **, p<0.01; ***, p<0.001 (unpaired t test). (**C**) Line charts representing the percent of CD5^+^ cells in CD4^+^ (left) and CD8^+^ (right) T-cell subsets at different time points post PHA, αCD3/CD28, PMA or rhIL2 exposure.


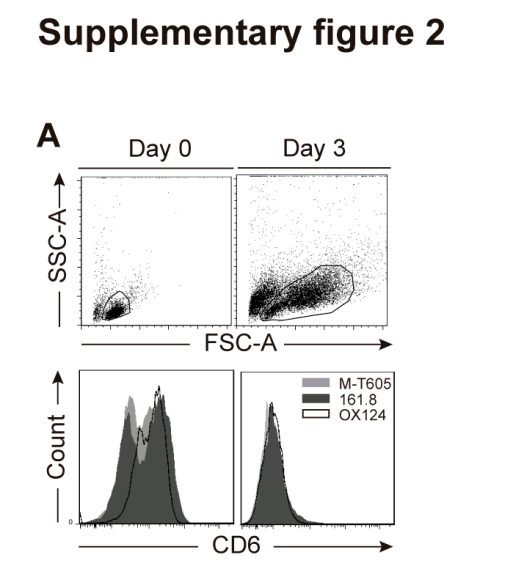


**Figure S2**. **Staining of PHA-activated PBLs with anti-D1 and anti-D3 CD6-specific mAbs.** PBLs (10^5^/well) cultured for 3 days in the presence of PHA (5 µg/mL) were analyzed by flow cytometry for CD6 surface expression with either FITC-labeled M-T605 mAb or unlabeled OX124 and 161.8 mAbs plus FITC-labeled goat anti-mouse Ig. Top, representative dot plots of the gating strategy used. Bottom, fluorescence histograms of the indicated anti-CD6 mAbs.


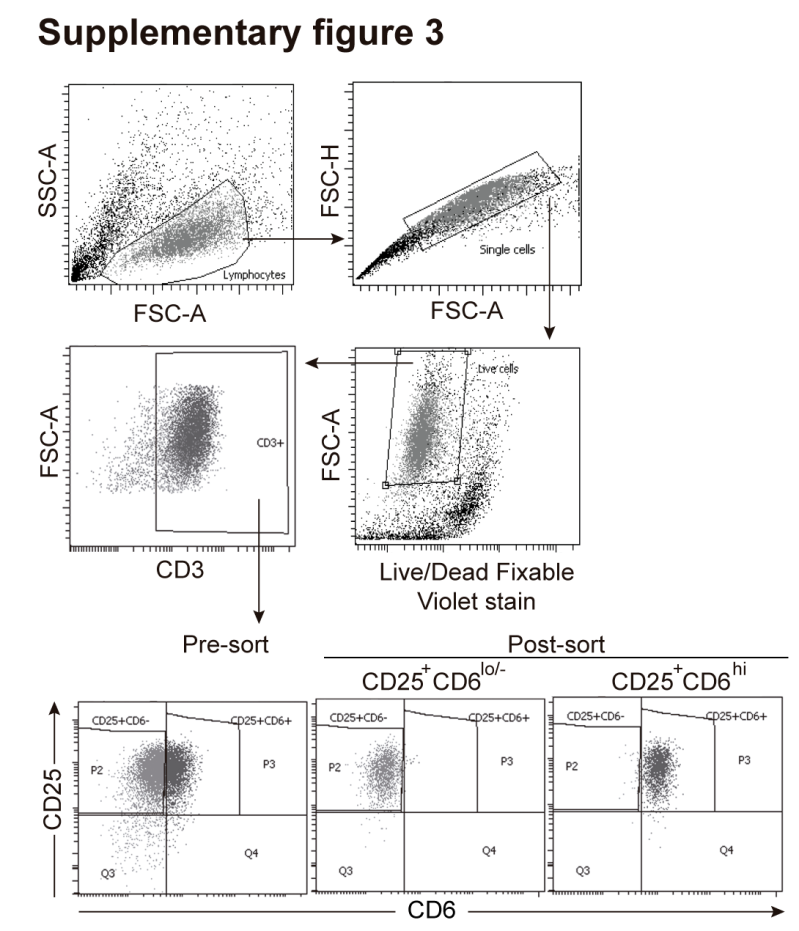


**Figure S3. Sorting strategy used for isolation of activated (CD25^+^) CD6^lo/-^ and CD6hi T-cells.** PBLs (5x10^7^ cells) cultured for 3 days in the presence of PHA (2 µg/mL) were stained for surface CD3 (UCHT1-PE), CD25 (MEM-181-APC) and CD6 (M-T605-FITC) expression, as well as with Live/Death violet staining for discrimination of viable/dead cells. CD25+CD6^lo/-^ and CD25+CD6hi cell subsets were sorted on a FACSAria instrument (BD Bioscience) following the indicated sequential gating strategy.


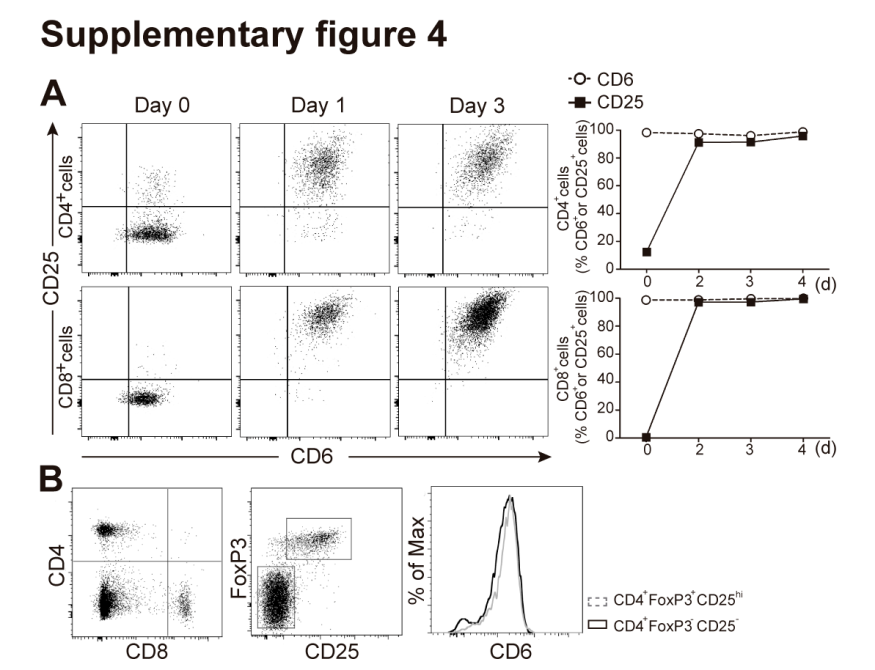


**Figure S4. Mouse CD6 surface expression in not down-regulated in T-cells with both activated and regulatory phenotype.** Splenocytes (2x10^5^) from C57BL/6J mice exposed to anti-CD3ε (145-2C11; 1 μg/mL; BD Pharmingen) plus anti-CD28 (37.51; 2 μg/mL; TONBO Bioscience) or rmIL-2 (10 ng/mL; ImmunoTools) for different time periods were then stained for CD4 (RM4-5-v450; BD Pharmingen), CD8 (53-6.7-PE-Cy5.5; Invitrogen), CD25 (PC61.5-FITC; BioLegend), and/or CD6 (OX-129-PE; BioLegend) surface expression. (**A**) **Left**, Representative dot plots of CD6 and CD25 expression on CD4^+^ (top panel) and CD8^+^ (bottom panel) mouse T-cells at day 0, 1 and 3 post-αCD3/CD28. **Right**, percent of CD6^+^ or CD25^+^ cells in CD4^+^ (top) and CD8^+^ (bottom) T-cells at different time points. Results are triplicates of one representative experiment from two performed. (**B**) Splenocytes from C57BL/6J mice were simultaneously stained for CD4, CD6, CD8 and CD25 surface expression and then for intracellular FoxP3 expression. **Left**, Dot plots illustrating the gating strategy used. **Right**, CD6 surface expression of gated CD4^+^FoxP3^+^ CD25^hi^ (dotted line) and CD4^+^FoxP3^-^ CD25^-^ (solid line) cells.
